# Supplementary material for: Direct prediction of gas adsorption via spatial atom interaction learning
Source: Nat Commun. 2023 Nov 3;14:7043. doi: 10.1038/s41467-023-42863-6 (PMC10624870; doi:10.1038/s41467-023-42863-6)
Supplement: Supplementary file 1 — Supplementary Information [file 41467_2023_42863_MOESM1_ESM.pdf]

Supporting Information for

Direct prediction of gas adsorption via spatial atom interaction learning

Jiyu Cui[1&], Fang Wu[2,3,4&], Wen Zhang[2&], Lifeng Yang[1,3&], Jianbo Hu[1,3], Yin Fang[2,3,5], Peng Ye[2,3,5], Qiang Zhang[2,3,5], Xian Suo[1,3], Yiming Mo[1,3], Xili Cui[1,3], Huajun Chen[2,3,5*], Huabin Xing[1,3*]

[1]Key Laboratory of Biomass Chemical Engineering of Ministry of Education, College of Chemical and Biological Engineering, Zhejiang University, Hangzhou 310012, China.

[2]College of Computer Science and Technology, Zhejiang University, Hangzhou 310027, China.

[3]Engineering Research Center of Functional Materials Intelligent Manufacturing of Zhejiang Province, ZJU-Hangzhou Global Scientific and Technological Innovation Center, Hangzhou 311215, China.

[4]School of Professional Studies, Columbia University, New York NY 10027, USA.

[5]Alibaba-Zhejiang University Joint Research Institute of Frontier Technologies, Hangzhou 310027, China.

[&]These authors contributed equally: Jiyu Cui, Fang Wu, Wen Zhang, Lifeng Yang.

[*]Corresponding author. E-mail: xinghb@zju.edu.cn; huajunsir@zju.edu.cn

Table of Contents

Supplementary Discussion 3

1. Related works 3

2. Model performance 5

3. Model interpretability 7

Supplementary Methods 8

4. DeepSorption network architecture 8

5. LSTM model architecture 11

6. Other deep learning models architecture 11

7. EKDL model architecture 11

8. GCMC simulations 12

9. Datasets 13

Supplementary Figures 15

Supplementary Tables 51

Supplementary Discussion

1. Related works

Recent years, due to the explosive growth in the number of crystalline porous materials, the speed of adsorption property characterization of crystalline porous materials has become a bottleneck that severely restricts the discovery of efficient crystalline adsorbents[1,2]. The emerged computational science 3rd paradigm, represented by molecular simulation technology, greatly alleviates this problem, but it still has an obvious trade-off between computing speed and accuracy, especially for highly polar gases, such as acetylene (C_2H_2) and carbon dioxide (CO_2)[3]. Machine learning techniques that can be used by domain experts in combination with massive data are thought to have sparked the "4th paradigm of science"[4]. In the current research on the adsorption performance prediction of crystalline porous materials, the descriptors of crystalline porous materials are used as the input, such as pore size, pore volume, accessible surface area, density and void fraction, which are generally developed and extracted from the raw structural data by means of computational science[5,6]. With the above-mentioned descriptors as the input, machine learning algorithms such as multilayer perceptron, random forest, decision tree and support vector machine algorithms are used to predict the adsorption performance[7-9]. Based on this idea, researchers have developed many new porous material descriptors by means of molecular simulation and calculation, such as secondary building unit (SBU)[10], element density[11], energy histogram[12], topology[6], etc., and have obtained good results in the prediction of methane and hydrogen storage capacities.

However, there are still many challenges with the above Expert-knowledge-driven learning (EKDL) method. One of the biggest challenges is that the loss of original information in the process of generating porous material descriptors, which would lead to low prediction accuracy of the model. Although the descriptors contain considerable expert knowledge, this is a one-way process from the original crystal structure to the generation of descriptors, and the original appearance of porous materials cannot be completely restored by the descriptors [5,13]. In addition, the method of EKDL has the drawback of descriptor extraction success rate. Since the extraction of descriptors is often not 100% successful, it will cause the inability of the model to predict the adsorption performance of some materials[11]. EKDL also encounters the problem of low prediction speed, as the descriptors calculation process is significantly slower than the subsequent machine learning prediction processes[5,14]. In addition, the method of descriptor extraction lacks good extensibility, for example, some models take secondary building units (SBUs) as input. However, since the number of SBUs is infinite in theory, the model cannot be used to predict its performance if the porous crystalline material to be predicted has SBUs that do not appear in the training set[13,15]. Although there are many descriptors that contain physical structure information and chemical element information, the separated input way of physical structure information and chemical element information can lead to serious fragmentation and loss of information, for example, functional sites in different distributions of porous materials always have distinctive influence on the adsorption performance[6,7,11,13].

These challenges limit the advances of machine learning in predicting the current adsorption performance for porous crystalline materials. The current successful models are basically applied in hypothetical MOFs (hMOF) dataset[3,13]. Relatively, it is still difficult to achieve good prediction performance, especially for strongly polar gases (e.g., acetylene and carbon dioxide) in the more complex Computation-Ready, Experimental MOF (CoREMOF) dataset (containing less than 12000 porous materials with up to 77 element types)[16].

In this study, DeepSorption realizes end-to-end prediction of adsorption properties by taking the

original information of crystalline porous materials (the atomic coordinates and element type information) as input to solve the problem of information loss. DeepSorption network shows good universality, expansibility and fast prediction speed. The Matformer model in DeepSorption network also realizes the in situ coupling input of spatial structure information and chemical element information, and shows benchmark adsorption prediction performance.

For deep learning, one of the emerging research field in machine learning, there have only been a few related studies for crystalline materials in recent years[17]. Currently, models based on graph neural networks have made great progress in the field of deep learning for crystalline materials[18-21]. Among them, the most representative method is the Crystal graph convolutional neural networks (CGCNN) method reported by Xie in 2017[21], which achieved excellent results on the prediction of formation energy, absolute energy, band gap properties and other properties of crystalline perovskite materials. Several new models based on graph neural networks inspired by CGCNN also obtained good results in the prediction of the properties of crystals with fewer atoms in a single cell (such as perovskite and drug crystals). However, the low R^2 (0.48) of CGCNN demonstrates that there is still considerable space for further improvement.

Furthermore, the Matformer model transfers the atomic coordinates and element type through 3D position encoding and Chemical element encoding modules respectively (Fig. S15). The Multi-scale Atom-attention mechanism is used to keep the spatial location information and the chemical element information of atoms when atoms interact with each other. As seen from the prediction results of the subsequent co-learning of expert knowledge (Table S4), DeepSorption is able to accurately extract the global structure information such as pore size, pore volume and specific surface area from the original material, which further contributes to the superior predicted performance of Matformer compared to those of graph neural network based models.

2. Model performance

Considering the importance of direct air capture and acetylene, methane and hydrogen storage technologies, CoREMOF (CO_2), hMOF (CO_2, N_2, H_2, CH_4) and EXPMOF (CO_2, C_2H_2) datasets were utilized to train the models. Besides the home-made Matformer and LSTM, the famous CGCNN and the traditional EKDL implemented by Multilayer Perceptron (MLP) are also employed as the contrast. As presented in Fig. 3 and Fig. S3, the predicted values and the true values are in good agreement on DeepSorption (Matformer+KCL) in hMOF-CO_2, hMOF-N_2 and CoREMOF-CO_2 tasks on the test set. Moreover, DeepSorption (Matformer+KCL) always shows more accurate predictions than other models (Matformer, LSTM+KCL, LSTM, CGCNN and EKDL models) in all examined cases with the highest coefficient of determination (R^2) and the lowest root mean squared error (RMSE) between the predicted adsorptions and the true adsorptions (Table S1-3).

Machine learning models based on handcrafted descriptors have achieved good results in molecular property prediction tasks, so it is very important and meaningful to use these descriptors as a comparison in the prediction of crystal porous materials property. Therefore, we used revised autocorrelation functions descriptors (RACs), many-body tensor representation descriptors (MBTR), smooth overlap of atomic positions descriptors (SOAP) and geometric structure descriptors for comparison. And the multi-layer perceptron (MLP) network was used as the machine learning model to predict the adsorption performance, and it was found that the effect of the models on CoREMOF dataset based on MBTR (MAE :21.54, R^2: 0.40) and RACs (MAE :18.64, R^2: 0.51) descriptors was better than that on geometric structure descriptors (MAE :21.78, R^2: 0.33), but still worse than that of DeepSorption (MAE :14.39, R^2: 0.70). And the model based on SOAP descriptors achieved the worst prediction results (MAE :30.05, R^2: -0.08), which may be due to the too long and sparse feature vector (with a length of 10560 on CoREMOF dataset). Meanwhile, it is worth mentioning that it takes a long time to calculate these descriptors on the CoREMOF dataset, especially for MBTR and RACs descriptors (about 7days and 5days, respectively). Since the atom number in a single cell of crystal porous materials (10^1~10^5) is often much larger than that in a single molecule (10^0~10^2), it further illustrates the advantage of the end-to-end model DeepSorption with atomic coordinates and element information as input in the prediction efficiency.

Both LSTM+KCL and DeepSorption show improved performance than above-mentioned EKDL models (including GEO_MLP, RAC_MLP, MBTR_MLP and SOAP_MLP) on all tasks (Table S1), demonstrating that the loss of structure information severely impairs machine learning performance. Both DeepSorption and Matformer outperform the LSTM+KCL and LSTM, especially on the complex CoREMOF-CO_2 tasks (Table S1). The RMSE decreased by an average of 17% on all tasks from LSTM to Matformer, which demonstrates the superiority of the Atom-attention mechanism in spatial atom interaction learning. Both on LSTM and Matformer, data-driven knowledge co-leaning model performs better than data-driven learning models on all tasks, which proves the further improvement in performance prediction for KCL models (Table S1-3). We further explore the prediction effect of expert knowledge on data-driven knowledge co-leaning models. Taking CoREMOF dataset as an example, we find Matformer based model can learn these knowledge (LCD, PLD, D, ASA, VF, AV) very well with high R^2 (0.90, 0.84, 0.97, 0.93, 0.95, 0.91, respectively) and low MAE (0.673, 0.573, 0.067, 218.274, 0.020, 0.049, respectively) (Table S4 and Fig. S3) in prediction on test set. The results indicate that DeepSorption, like human scientists, could well learn and utilize this information, which also explains why data-driven knowledge co-leaning models can achieve a better learning effect compared to the solely data-driven learning methods. In contrast,

both LSTM and CGCNN cannot accurately predict expert knowledge on CoREMOF dataset (Table S5, 6 and Fig. S9, 12), we speculate that the input method of LSTM and CGCNN cannot make good use of the spatial coordinate information of materials, which leads to their poor adsorption prediction performance. Considering that prediction effect of LSTM and LSTM+KCL is better than that of CGCNN, we believe that the mechanism of LSTM is still very helpful to the model to mine the elemental chemical information of materials. For EXPMOF dataset, in order to represent the results of DeepSorption more clearly, the experimental (blue) and predicted (red) adsorption isotherms of each material in EXPMOF-CO_2 (Fig. S16-21) and EXPMOF-C_2H_2 (Fig. S22-28) are drawn separately.

In order to better handle the periodicity of the crystalline material, we considered periodicity when calculating the distance matrix in the multi-scale atom-attention module, and used the periodicity to perform data augmentation through cell expansion, and achieved some improvement in the prediction effect (R^2: 0.656 vs 0.701, MAE: 15.35 vs 14.39) (Table S9). However, it is worth mentioning that the periodic calculation of distance matrix will greatly increase the calculation time, which consumes more time than the original method of distance matrix calculation by torch.cdist function.

In order to further enhance invariance ability to the rotation and translation of the DeepSorption model, we use the crystal data augmentation (translation, rotation and extended cell) methods reported in the literature[17] to train the perception of rotation and migration invariance of positional encoding module in the model. By analyzing the prediction results of the model trained by the data enhancement method on the test set data after translation and rotation, we found that both the translation and rotation of the crystal data did not produce a large deviation of the prediction results (MAE :15.126~15.508, R^2: 0.662~0.642) (Table S11).

3. Model interpretability

The interpretability of deep learning models has always been a focus of researchers, especially in the field of 'AI for Science'. Exploring the interpretability of models with excellent predictive performance is an important means to ensure that models really learn knowledge and gain chemical insight. Machine learning models with porous material descriptors as input can judge the importance of different expert knowledge descriptors on adsorption performance by analyzing the weight of different descriptors in the model, but this method cannot be applied to the deep learning algorithm based on raw data. Fortunately, the Multi-scale Atom-attention mechanism allows the model to be interpretable at atomic scale. With the help of the Multi-scale Atom-attention mechanism, the interpretability at different scales is presented in this study. Weights are extracted from Atom-attention layer, and atom pair interactions with higher weight ranking are displayed by connecting lines to show the atomic interactions that the model concentrates more on. Taking 5Å distance bar scale as an example, we first drawn the structure of crystalline porous material by using atomic coordinate and chemical element type information. Then, the Atom-attention weight parameters for each atom pair of DeepSorption with distance bar of 5Å are extracted and sorted from largest to smallest. The top 20 atomic pairs in weight order of each layer of DeepSorption with distance bar of 5Å are highlighted with red lines. Besides SIFSIX-1-Cu, we also calculated and demonstrated 3D attention visualization for several other representative MOF materials, including MFM-188 (Fig. S33), MOF-505 (Fig. S34), HKUST-1 (Fig. S31), UTSA-74 (Fig. S32). We also provide a display version in the form of HTML files in the attachment for readers to freely change perspectives for better display the interaction of atomic attention mechanism in 3D space (https://doi.org/10.5281/zenodo.7699719).

Supplementary Methods

4. DeepSorption network architecture

a. Matformer model architecture

Input. A crystalline material $S = (E, P)$ has N atoms and C element types, where $E \in \mathbb{R}^{N\times C}$ represents atoms, and each row e_i represents one atom with its type encoded, and $P \in \mathbb{R}^{N\times 3}$ represents 3D coordinates of the atoms that each row $p_i = [p_i^x, p_i^y, p_i^z]$.

Chemical element encoding. Instead of randomly assigning a representation to each atom, we incorporate chemical element knowledge graph (KG)[22] (Fig. S2) to initialize the embedding of each atom. This Chemical Element KG collect the fundamental chemical domain knowledge from the Periodic table of Elements. Since each element contains more than 15 attributes, (e.g., metallicity, periodicity, state, weight), KG models the specified relations between elements and attributes in the form of triple (head entity, relation, tail entity) abbreviated as (h, r, t), for example (Gas, isStateOf, Cl). In order to capture the semantics of each element, we use RotateE[23] which defines each relation as a rotation in the complex vector space to train Chemical Element KG. The score function of (h, r, t) is formulated as follows:

$$\mathrm{f(h, r, t)} = ||\mathrm{h} \circ \mathrm{r} - \mathrm{t}|| \quad (1)$$

where $\mathrm{h, r, t}$ denote the embedding of head entity, relation and tail entity respectively, and $\circ$ is the Hadmard product. After that, we obtain the embedding of each entity and relation in the KG. We pick out the embeddings of these element entities as the initial representations of the corresponding atoms. In this way, information in Chemical Element KG is encoded in the initial features.

Element knowledge graph embedding. We crawl all the chemical elements and their attributes from the periodic table of elements. Each element contains more than 15 attributes, including metallicity, periodicity, state, weight, electronegativity, electron affinity, melting point, boiling point, ionization, radius, hardness, modulus, density, conductivity, heat, and abundance. The extracted triples in the form of (h, r, t) are constructed in KG, indicating the relationship between elements and attributes.

3D position encoding. In order for the model to take advantage of the 3D coordinates of atoms, it is crucial to encode the positional information correctly. To this end, we leverage "3D positional encoding" to the input embeddings. The positional encodings have the same dimension d_{model} as the embeddings. Specifically, we map coordinates $p_i = [p_i^x, p_i^y, p_i^z]$ of each axis into $\left(PE_{p_i^x}, PE_{p_i^y}, PE_{p_i^z}\right)$ using sine and cosine functions of different frequencies:

$$PE_{(p_i^x,2j)} = \sin\left(\frac{\lambda p_i^x}{10000^{2j/d_{model}}}\right) \quad (2)$$

$$PE_{(p_i^x,2j+1)} = \cos\left(\frac{\lambda p_i^x}{10000^{2j/d_{model}}}\right) \quad (3)$$

$$PE_{(p_i^y,2j)} = \sin\left(\frac{\lambda p_i^y}{10000^{2j/d_{model}}}\right) \quad (4)$$

$$PE_{(p_i^y,2j+1)} = \cos\left(\frac{\lambda p_i^y}{10000^{2j/d_{model}}}\right) \quad (5)$$

$$PE_{(p_i^z,2j)} = \sin\left(\frac{\lambda p_i^z}{10000^{2j/d_{model}}}\right) \quad (6)$$

$$PE_{(p_i^z,2j+1)} = \cos\left(\frac{\lambda p_i^z}{10000^{2j/d_{model}}}\right) \quad (7)$$

where p_i^x is the position on x-axis, p_i^y is the position on y-axis, p_i^z is the position on z-axis, and j is the dimension. That is, each dimension of the positional encoding corresponds to a sinusoid or cosinusoid. The hyperparameter λ is multiplied to avoid the great variance per position across dimensions. These embeddings are merged with atomic embeddings to obtain a joint representation of each atom $x_i = e_i + \text{Linear}(PE_{p_i^x}|PE_{p_i^y}|PE_{p_i^z})$, and fed into the Multi-Scale Atom-attention module.

MSA. In order to recognize the interactions between atoms at different scales, we design a Multi-scale Atom-attention (MSA), which is a scale-aware multi-head attention mechanism (Fig. S4). Its input is a sequence of atom vectors, and the output is a sequence of updated atom vectors in the same order as the input. For each attention head, we assign a visible distance τ_s to make atoms within τ_s visible to each other during attention calculation in this head. With the input atom representation sequence $(x_1, x_2, x_3, \dots, x_i, \dots, x_N)$, each head of MSA first generates a key, value and query based on each atom vector $\boldsymbol{x}_i$:

$$q_i = \text{f}_\text{Q}(x_i) \quad (8)$$

$$k_i = \text{f}_\text{K}(x_i) \quad (9)$$

$$v_i = \text{f}_\text{V}(v_i) \quad (10)$$

where q_i, k_i, v_i are query, key and value vector with dimension d_k. Then an attention score is calculated based on the similarity between query q_i to key of atoms whose distance to atom i is within τ_s. Specifically, the attention that the atom i pays to j can be formulated as:

$$a_{ij}^{\tau_s} = \frac{q_i k_j^T \cdot 1_{\{d_{ij}<\tau_s\}}}{\sqrt{d_k}} \quad (11)$$

where $d_{ij} = \|p_i - p_j\|_2$ is the Euclidean distance between atom i and j, $1_{\{d_{ij}<\tau_s\}}$ is the indicator function that makes the score between two atoms beyond distance τ_s to 0, and $\frac{1}{\sqrt{d_k}}$ is a scaling factor. The output vector of atom i at this attention head is:

$$z_i^{\tau_s} = \sum_{j=1}^{N} \sigma(a_{ij}^{\tau_s}) v_j \quad (12)$$

here σ denotes the softmax function. For each attention head, we specify distinct distances to enable the model to capture knowledge at different scales. Then vectors of atom i from different heads are concatenated resulting a multi-scale vector $\boldsymbol{z}_i$, followed by a feed-forward network to map it to dimension d_{model}.

Feed-forward neural network (FNN). Additional to the multi-head attention, a fully connected feed-forward neural network is added to each Multi-scale Atom-attention layer, which is applied to each position separately and identically. The full transformation including the residual connection can be formulated as:

$$\text{FNN}(z) = \max(0, z\mathbf{W_1} + \mathbf{b_1})\mathbf{W_2} + \mathbf{b_2} \quad (13)$$

$$z = \text{LayerNorm}(z + \text{FNN}(z)) \quad (14)$$

where W and b are learnable weight matrix and bias parameters, respectively.

b. KCL

After having the atom representations $\{\boldsymbol{z}_i\}_{i=1,\dots,N}$, we feed them into a fully connected layer to conduct prediction. The output dimension of the layer is depended on the number of predicted targets. That is, if we apply knowledge co-learning tasks and predict the values of n targets (e.g., LCD, PLD, ASA) simultaneously during the training phase, the output will be a vector of dimension

n. For CoREMOF dataset, the knowledge co-learning tasks are LCD, PLD, D, ASA, VF and AV, and the adsorption task is AD_{CO2} (adsorption uptake of CO_2), thus the output is a vector of dimension 7. For hMOF dataset, the knowledge co-learning tasks are LCD, PLD, D, ASA and VF, and the adsorption tasks are AD_{CO2}, AD_{N2} (adsorption uptake of N_2), thus the output is a vector of dimension 7. Otherwise, we also only focus on 1 target (adsorption uptake) in training, and the dimension of the output will be 1.

c. Hyper-parameter

Our Matformer has 6 Multi-scale Atom-attention layers with 4 attention heads. Each head is assigned a scale, and we set the distance bar as {5Å, 8Å, 12Å, ∞} or {3Å, 5Å, 8Å, ∞} for different tasks. λ in 3D position encoding is kept as 10. We use ReLU as the activation function and a dropout rate of 0.1 for each layer. The dimensionality of input and output is d_{model}=512 and the inner-layer has dimensionality 2048. The query, key and value are with dimension $d_k = 128$. Our model is implemented with PyTorch. We develop all codes on a Ubuntu Server with 4 GPUs (NVIDIA GeForce 1080Ti). We use the Adam optimizer with an initial learning rate of 0.0005 and batch size of 16. We truncate the length of crystals with a maximum length of 1024. We use grid search to tune the hyper-parameters of our model and baselines based on the validation dataset.

5. LSTM model architecture

Long Short-Term Memory (LSTM)[24] model is a classical recurrent neural network that is specially designed to solve the long-term dependence problem in Natural Language Processing (NLP) field.

In LSTM, the forgetting gate, input gate, and output gate of each block determine how much of the previous state is retained to current state, how much of the input is retained to the state, and which states are output, respectively. As presented in Fig. S5, we also utilize LSTM model for the adsorption performance prediction of serialized crystalline porous materials which is sorted by distance to the origin. We extend the unit cell to the positive and negative directions in the three directions of Cartesian 3D coordinates X, Y, and Z, and obtain the spatial information as well as element type of the N neighboring atoms nearest to each atom, which is inputted to model together with Cartesian coordinates and element types of the center atom. We also input the van der Waals radius V of the atom and the distance of each atom from the origin D_a as supplementary information. To make the model with the ability to deal with different numbers of nearest atoms and learn the atoms embedding better, we deploy a feed-forward neural network converting the spatial and chemical information of central atom and its neighbors into a virtual atom, whose embedding represents the aggregated chemical property. Then the virtual atom embedding is combined with the spatial information of the center atom, which is inputted to LSTM to predict the adsorption properties. Our LSTM model has 2 layers and inner-layer dimension is 128. The elements embedding encoding are vectors with dimension 40 and aggregate 5 atoms which are nearest to the center atom. Our model is implemented with PyTorch. We develop all codes on a Ubuntu Server with 4 GPUs (NVIDIA GeForce 1080Ti). We use the Adam optimizer with an initial learning rate of 0.0005 and batch size of 16. We truncate the length of crystals with a maximum length of 1024. We use grid search to tune the hyper-parameters of our model and baselines based on the validation dataset.

6. Other deep learning models architecture

Crystal graph convolutional neural networks (CGCNN) is a deep learning network developed by Xie[21], which could learn material properties of the crystalline materials and provide a universal and interpretable representation of crystalline materials. The main idea in this approach is to represent the crystal structure by a crystal graph that encodes both atomic information and bonding interactions between atoms, and then build a convolutional neural network on top of the graph to automatically extract representations. For the hyper-parameter, the maximum number of neighbors while constructing the crystal graph is 8, the cutoff radius for searching neighbors is 8, the minimum distance for constructing Gaussian Distance is 0, step size for constructing Gaussian Distance is 0.2, number of bond features is 64, number of hidden atom features in the convolutional layers is 3, number of hidden atom features in the convolutional layers is 128, number of hidden layers after pooling is 1.We split the datasets with a ratio for train/validation/test as 0.7:0.15:0.15 in the CoREMOF and hMOF dataset. We trained all codes on a Ubuntu Server with 1 GPUs (NVIDIA GeForce 3090Ti). CGCNN is trained to minimize the MSE loss, which is the mean overseen data of the squared differences between true and predicted values. We used the Adam optimizer with an initial learning rate of 0.01 and batch size of 256.

MOFNet[25] is an interpretable graph transformer network for predicting adsorption isotherms of MOFs. The prediction of MOFNet originates from both local representation and global representation. The number of graph transformer network layers was set to 2 and the hidden state

of each layer was set to 1024. We applied a three-layer MLP as the global feature encoder, and the dimensions of these three layers were set to 128, 512, and 1024. The model was trained for 300 epoch using a batch size of 32 MOF structures. We split the datasets with a ratio for train/validation/test as 0.7:0.15:0.15 in the CoREMOF dataset. We trained all codes on a Ubuntu Server with 1 GPUs (NVIDIA GeForce 3090Ti). MOFNet is trained to minimize the MSE loss, which is the mean overseen data of the squared differences between true and predicted values. We used the Adam optimizer with $\beta 1 = 0.9$, $\beta 2 = 0.98$, and $\epsilon = 10^{-9}$.

7. EKDL model architecture

Expert-knowledge-driven learning (EKDL) based on descriptors is the most popular machine learning methods in adsorption prediction task[26-27]. The descriptors, including geometric structure descriptors, revised autocorrelation functions descriptors (RACs)[28], many-body tensor representation descriptors (MBTR)[29] and Smooth overlap of atomic positions descriptors (SOAP)[30], with expert knowledge generated by high-throughput analysis tool are utilized as input. For models based on geometric models, the descriptors are LCD (largest cavity diameter), PLD (pore limiting diameter), D (density), ASA (accessible surface area), VF (void fraction), AV (accessible volume). For hMOF dataset, the descriptors are LCD (largest cavity diameter), PLD (pore limiting diameter), D (density), ASA (accessible surface area), VF (void fraction), which are calculated using Zeo++ programs (version: 0.3) with radius_of_area_probe of 1.655, area_monte_carlo_samples of 2000 and porosity_monte_carlo_samples of 100000. The RACs descriptors are calculated using Molsimplify packages[31](version: 1.7.2). The MBTR descriptors are calculated using MBTR package[29] (version: 0.0.1). The SOAP descriptors are calculated using DScribe package[32] (version: 2.0.1) with rcut of 8, nmax of 8, lmax of 6, σ of 0.2 and average method of inner average. The machine learning algorithm is Multilayer Perception (MLP). The MLP model is 2 layers with layer dimension of 512 and 1024 respectively. We split the datasets with a ratio for train/validation/test as 0.7:0.15:0.15 in the CoREMOF and hMOF dataset. We trained all codes on a Ubuntu Server with 1 GPUs (NVIDIA GeForce 3090Ti). EKDL is trained to minimize the MSE loss, which is the mean overseen data of the squared differences between true and predicted values. We used the Adam optimizer with an initial learning rate of 0.0002 and batch size of 1024.

8. GCMC simulations

All the GCMC simulations were performed in Material Studio 2017R2 package. The crystal structure of the SIFSIX-2-Cu-i, Zn-MOF-74, and ZJNU-103 were chosen after the DFT geometry optimization. The framework and the individual C_2H_2 and CO_2 were considered to be rigid during the simulation. The charges for atoms of the SIFSIX-2-Cu-i, Zn-MOF-74, and ZJNU-103 were derived from QEq method and QEq charged. The simulations adopted the adsorption isotherms task at 298 K, Metropolis method in sorption module and the universal force field (UFF). The charge of the C_2H_2 and CO_2 were also derived from the QEq method. The interaction energy between the adsorbed molecules and the framewrok were computed through the Coulomb and Lennard-Jones 6-12 (LJ) potentials. The cutoff radius was chosen 18.5 Å for LJ potential and the long range electrostatic interactions were handled using the Ewald summation method. The loading steps and the equilibration steps were 1×10^7, the production steps were 1×10^7. It's worth noting that the simulated values through GCMC for weakly polar gases such as nitrogen, hydrogen and methane are in good agreement with the experimental values, but for strongly polar gases such as alkynes and olefin gases, the simulated values may differ greatly from the experimental values.

9. Datasets

CoREMOF dataset:

CoREMOF (Computation-Ready, Experimental MOF)[16] dataset includes over 11000 computation-ready, experimental three-dimensional metal-organic frameworks (MOFs) that contains more than 77 elements. MOFs, a kind of crystalline materials, are widely used in gas storage, separation, catalysis, molecular censor, and drug release. CoREMOF is obtained from Cambridge Structural Dataset and Web of Science search. The coordinates with low partial occupancies were moving and the structure were converting to P1 symmetry to improve the quality of the dataset. The CoREMOF dataset includes more than 350 topologies and more than 80 of these topologies are represented by 10 or more structures. The five most common topologies are pcu (26.7%), dia (12.8%), rtl (4.1%), ths (3.9%), and bcu (3.7%). The dataset contains 3D Cartesian coordinates and the corresponding element types of MOFs, and the corresponding adsorption uptake (unit: $cm^3\ g^{-1}$) at 1bar and 298 K of carbon dioxide (CO_2) determined by Grand canonical Monte Carlo (GCMC) simulations[33], and their corresponding expert knowledge, including LCD (largest cavity diameter, unit: Å), PLD (pore limiting diameter, unit: Å), D (density, unit: $cm^3\ g^{-1}$), ASA (accessible surface area, unit: $m^2\ g^{-1}$), VF (void fraction, unit: None), AV (accessible volume, unit: $cm^3\ g^{-1}$) (Fig. S6). The correlation coefficient matrix heatmap indicates that although the correlation coefficient of expert knowledge with each other is very high, the correlation coefficient between expert knowledge and CO_2 uptake is very low (LCD-AD_{CO2}:-0.14, PLD-AD_{CO2}:-0.12, D-AD_{CO2}:-0.02, ASA-AD_{CO2}:-0.05, VF-AD_{CO2}:0.00, AV-AD_{CO2}:-0.11), suggesting that the accurate prediction of CO_2 uptake is not sufficient based on expert knowledge alone (Fig. S1).

hMOF dataset:

hMOF dataset includes over 300,000 hypothetical MOFs containing 16 elements, which is constructed with the ToBasCCo program[3]. MOFs in hMOF dataset are built with rigid organic and inorganic struts called secondary building units (SBU)s. MOFs in hMOF dataset include up to 95 kinds of organic secondary building units (SBUs) and 8 kinds of metal SBUs used in the generation. 19 kinds of functional groups are used to decorate the unfunctionalized hypothetical MOFs. MOF dataset includes 3D Cartesian coordinates and the corresponding element types of MOFs, and corresponding adsorption uptake (unit: mmol g^{-1}) at 0.15 bar and 298 K of carbon dioxide (CO_2), adsorption uptake (unit: mmol g^{-1}) at 0.85 bar and 298 K of nitrogen (N_2) determined by Grand canonical Monte Carlo (GCMC) simulations, and their corresponding expert knowledge, including LCD (largest cavity diameter, unit: Å), PLD (pore limiting diameter, unit: Å), D (density, unit: $cm^3\ g^{-1}$), ASA (accessible surface area, unit: $m^2\ g^{-1}$), VF (void fraction, unit: None) (Fig. S7).

EXPMOF dataset:

The EXPMOF dataset constructed by ourselves is composed of EXPMOF-CO_2 and EXPMOF-C_2H_2. Different from CoREMOF and hMOF dataset, the data of EXPMOF dataset is from experiments. The MOFs includes UTSA series, CPL series, MFM series, MOF-74 series, SIFSIX series, HKUST series, SNNU series, UPC series, NPU series, NTU series, HKUST series, FJU series, FJI series, MECS series, NUC series, ELM series, ZJNU series, ZJU series, and etc. The 3D Cartesian coordinates and the corresponding element types are collected from reported literature or our lab. EXPMOF-CO_2 contains 112 data, and EXPMOF-C_2H_2 contains 140 data. In more details, adsorption isotherms are extracted from the figures of literature and then are interpolated for data

alignment. The adsorption uptake data are collected at 0-1 bar and 295-298K. The expert knowledge of MOFs, including LCD, PLD, D, ASA and AV, are calculated using Zeo++ programs.

Supplementary Figures

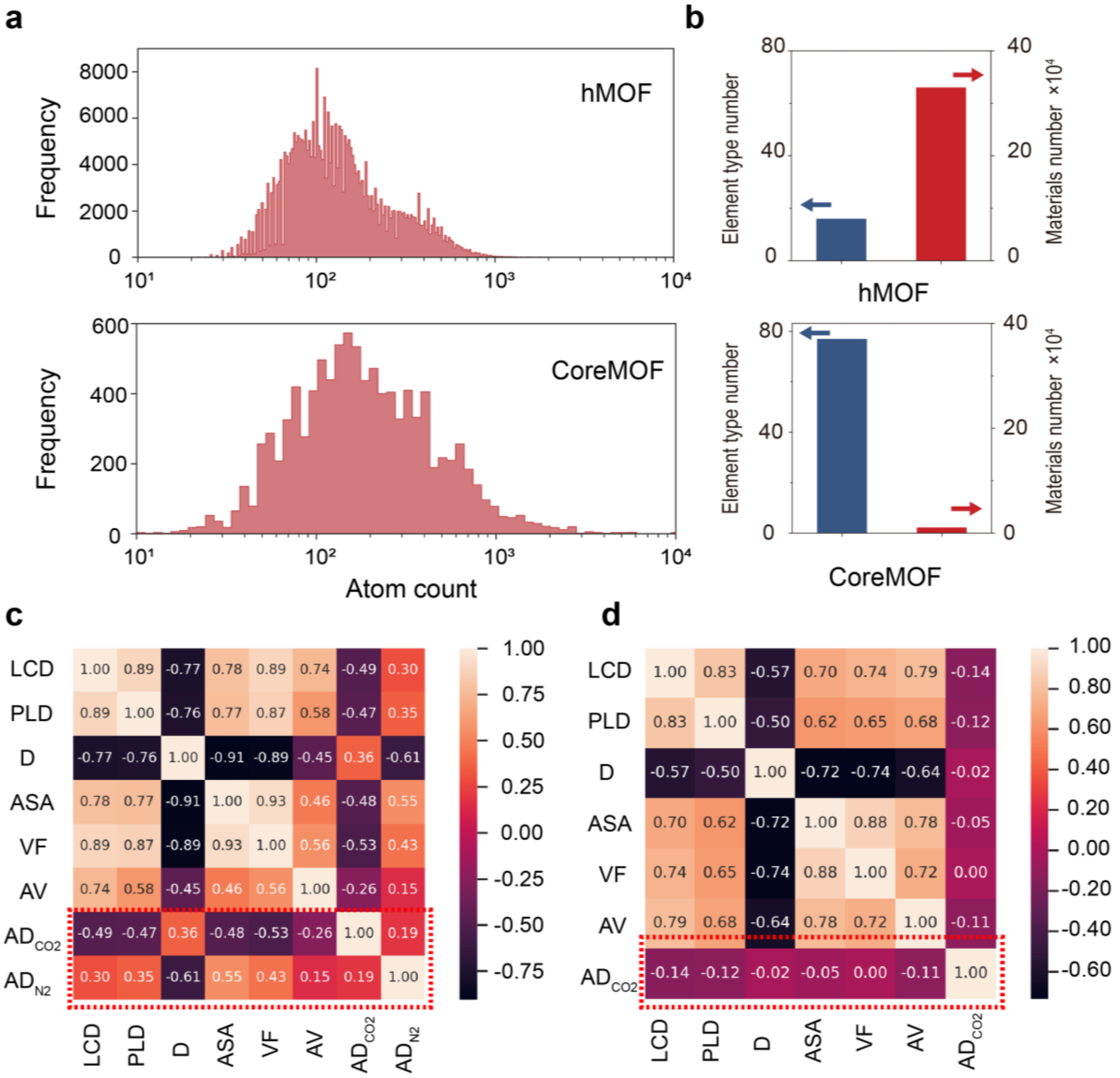

Fig. S1 | Data analysis of hMOF and CoREMOF datasets. a, The distribution of atomic number in single crystal cells of MOFs in CoREMOF and hMOF datasets. **b**, The material type number and element type number of CoREMOF and hMOF datasets. **c**, The correlation coefficient matrix heatmap of LCD (largest cavity diameter), PLD (pore limiting diameter), D (density), ASA (accessible surface area), VF (void fraction), AV (accessible volume), AD_{CO2} (adsorption uptake of CO_2) and AD_{N2} (adsorption uptake of N_2) in hMOF dataset. **d**, The correlation coefficient matrix heatmap of LCD, PLD, D, ASA, VF, AV, AD_{CO2} in CoREMOF dataset.

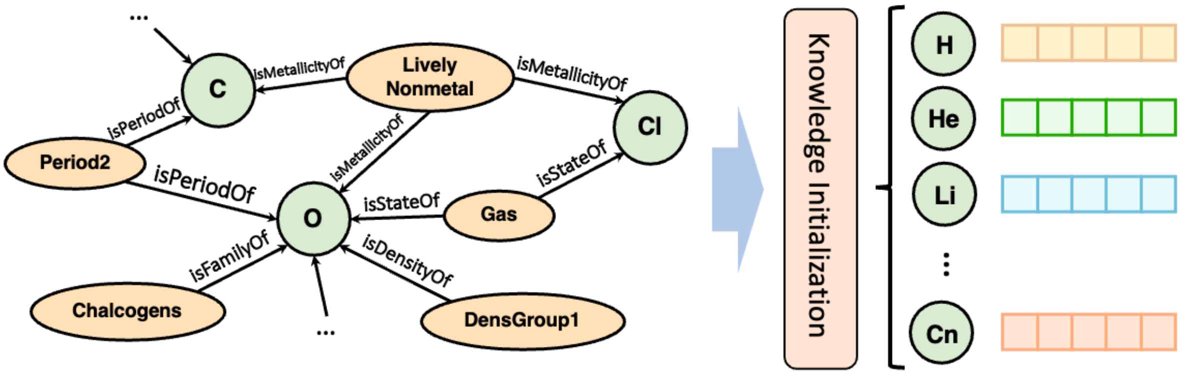

Fig. S2 | Chemical Element Knowledge Graph. Chemical Element Knowledge Graph is incorporated to initialize the embedding of each atom according to its element type. This Chemical Element Knowledge Graph has collected the fundamental chemical domain knowledge from the Periodic table of Elements.[22]

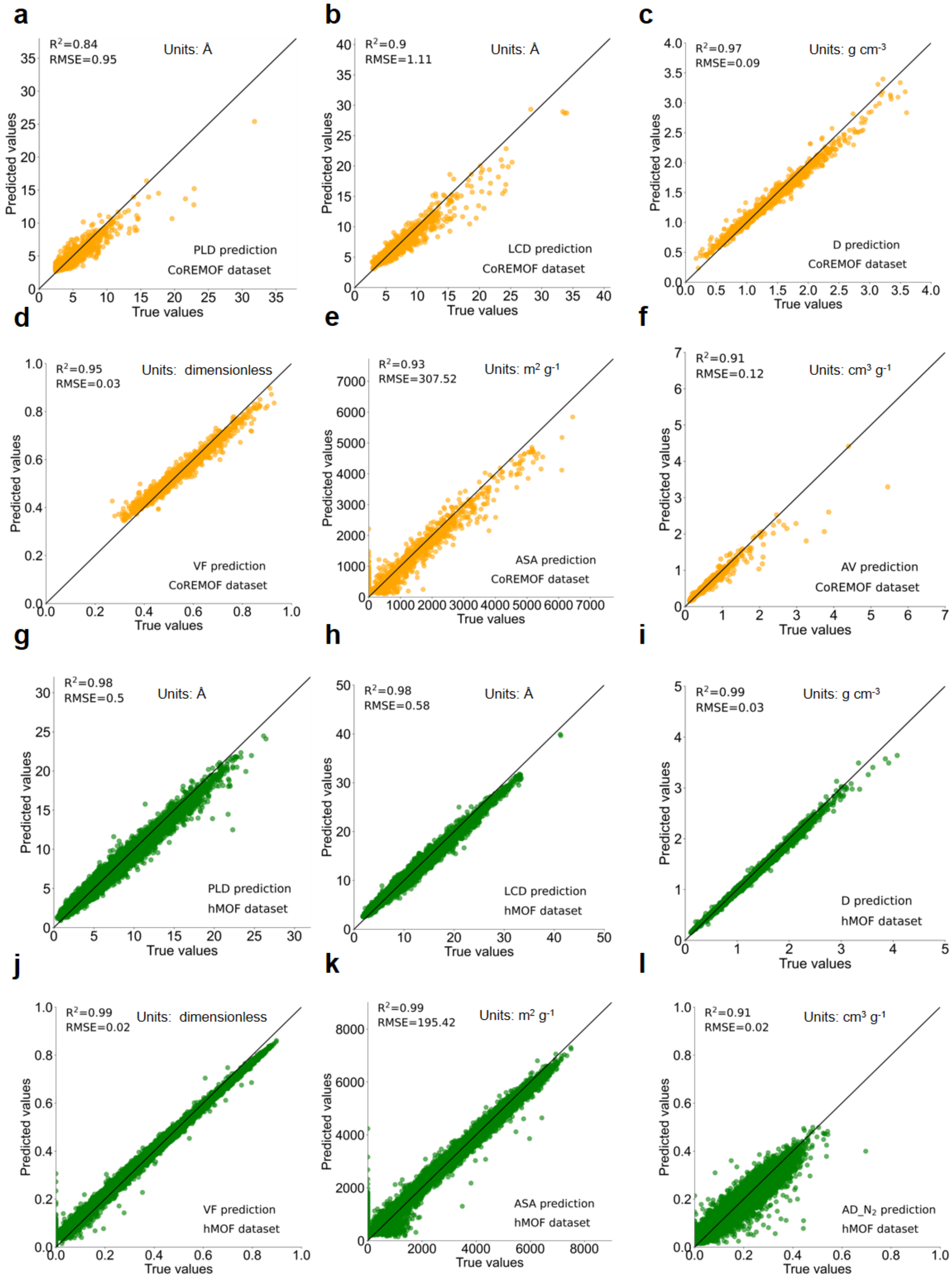

Fig. S3 | Prediction performance of DeepSorption on CoREMOF and hMOF datasets. a, The correlations between true values and predicted values of PLD (pore limiting diameter) on test set using DeepSorption on CoREMOF dataset. **b,** The correlations between true values and predicted values of LCD (largest cavity diameter) on test set using DeepSorption on CoREMOF dataset. **c,**

The correlations between true values and predicted values of D (density) on test set using DeepSorption on CoREMOF dataset. **d,** The correlations between true values and predicted values of VF (void fraction) on test set using DeepSorption on CoREMOF dataset. **e,** The correlations between true values and predicted values of ASA (accessible surface area) on test set using DeepSorption on CoREMOF dataset. **f,** The correlations between true values and predicted values of AV (accessible volume) on test set using DeepSorption on CoREMOF dataset. **g,** The correlations between true values and predicted values of PLD (pore limiting diameter) on test set using DeepSorption on hMOF dataset. **h,** The correlations between true values and predicted values of LCD (largest cavity diameter) on test set using DeepSorption on hMOF dataset. **i,** The correlations between true values and predicted values of D (density) on test set using DeepSorption on hMOF dataset. **j,** The correlations between true values and predicted values of VF (void fraction) on test set using DeepSorption on hMOF dataset. **k,** The correlations between true values and predicted values of ASA (accessible surface area) on test set using DeepSorption on hMOF dataset. **l,** The correlations between true values and predicted values of AD_N_2 (N_2 adsorption capacity) on test set using DeepSorption on hMOF dataset.

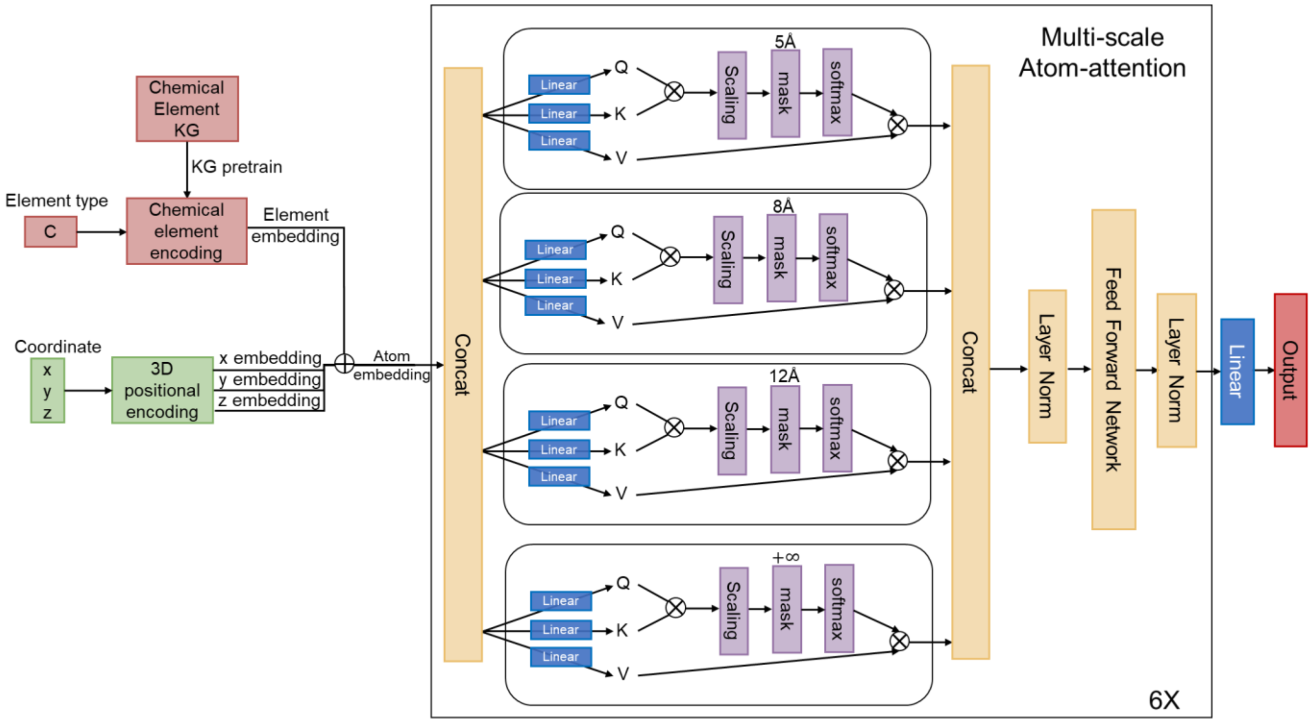

Fig. S4 | Matformer model architecture. Component modules and flow of the Matformer model, including the chemical element encoding modules, 3D positional encoding modules, and the Multi-scale Atom-attention module.

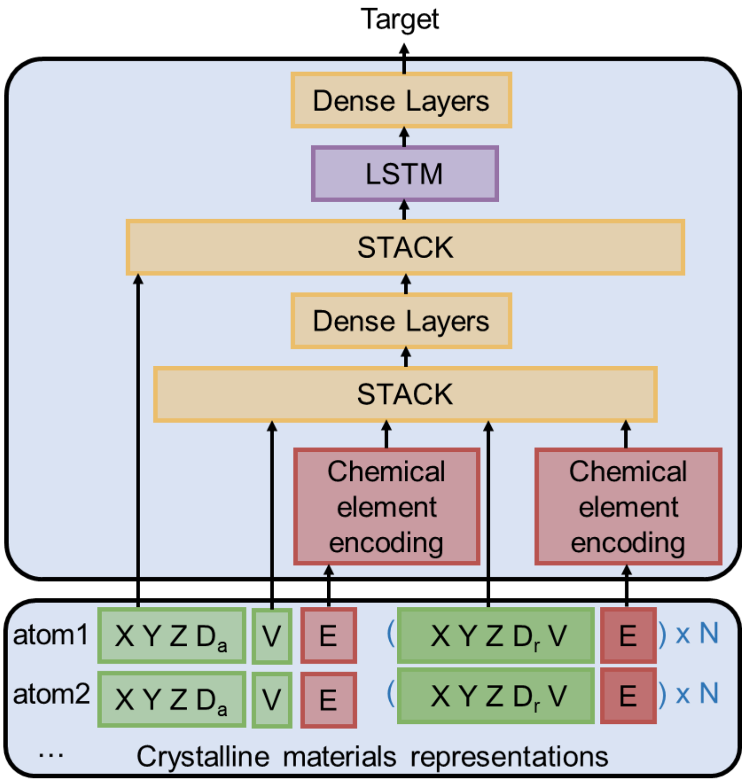

Fig. S5 | Scheme of Long Short-Term Memory model used for adsorption performance prediction of crystalline materials. X, Y, Z is the Cartesian coordinates of atoms; E is the element types of atoms; D_a is the absolute distance of the atom derived from the raw structure; D_r is the relative distance of other atoms to the central atom; V is the van der Waals radius of atoms.

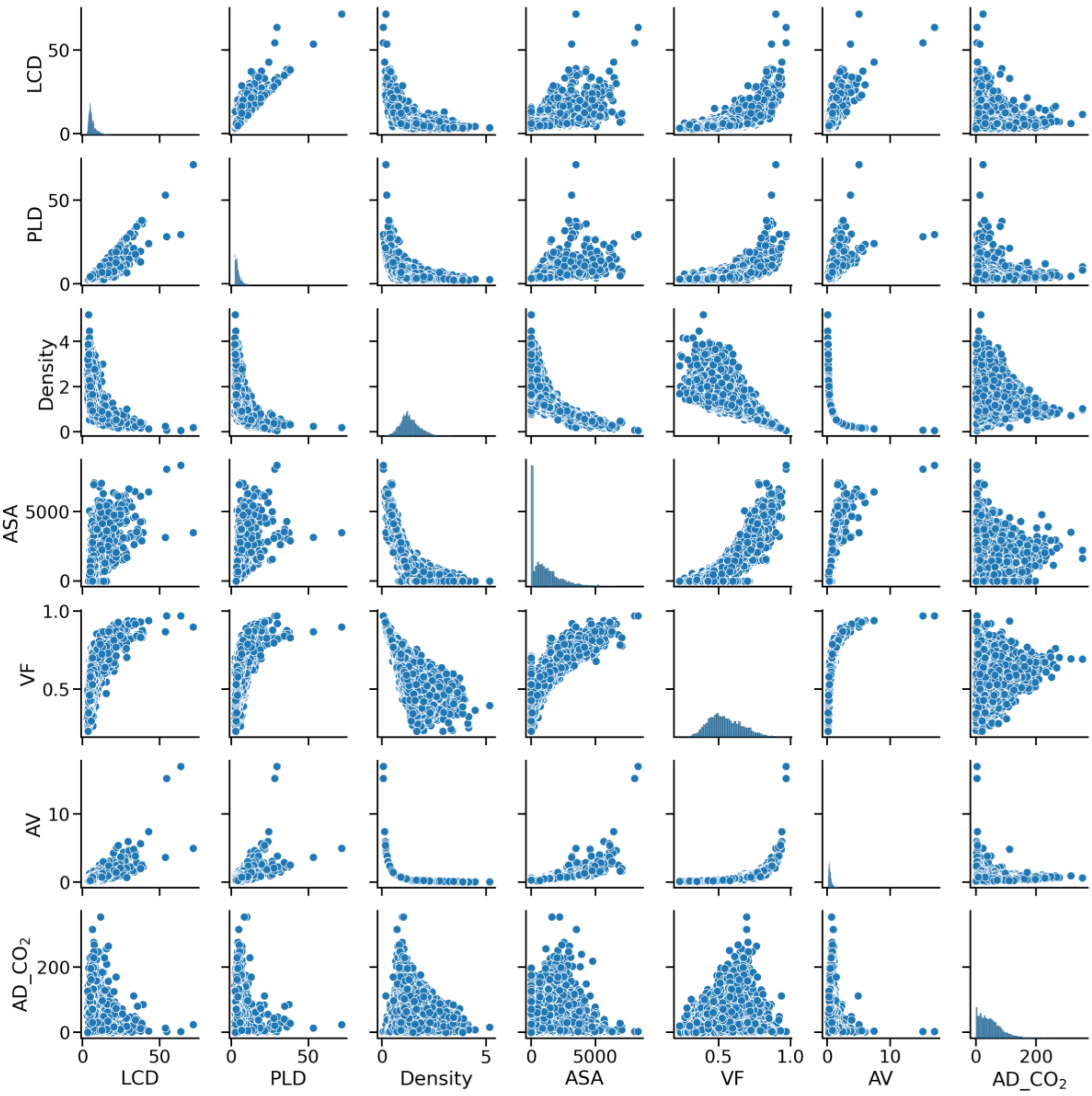

Fig. S6 | Data analysis of CoREMOF dataset. The relationship between LCD (largest cavity diameter), PLD (pore limiting diameter), Density, ASA (accessible surface area), VF (void fraction), AV (accessible volume) and AD_CO_2 (CO_2 adsorption capacity) in CoREMOF dataset by means of scatter plot.

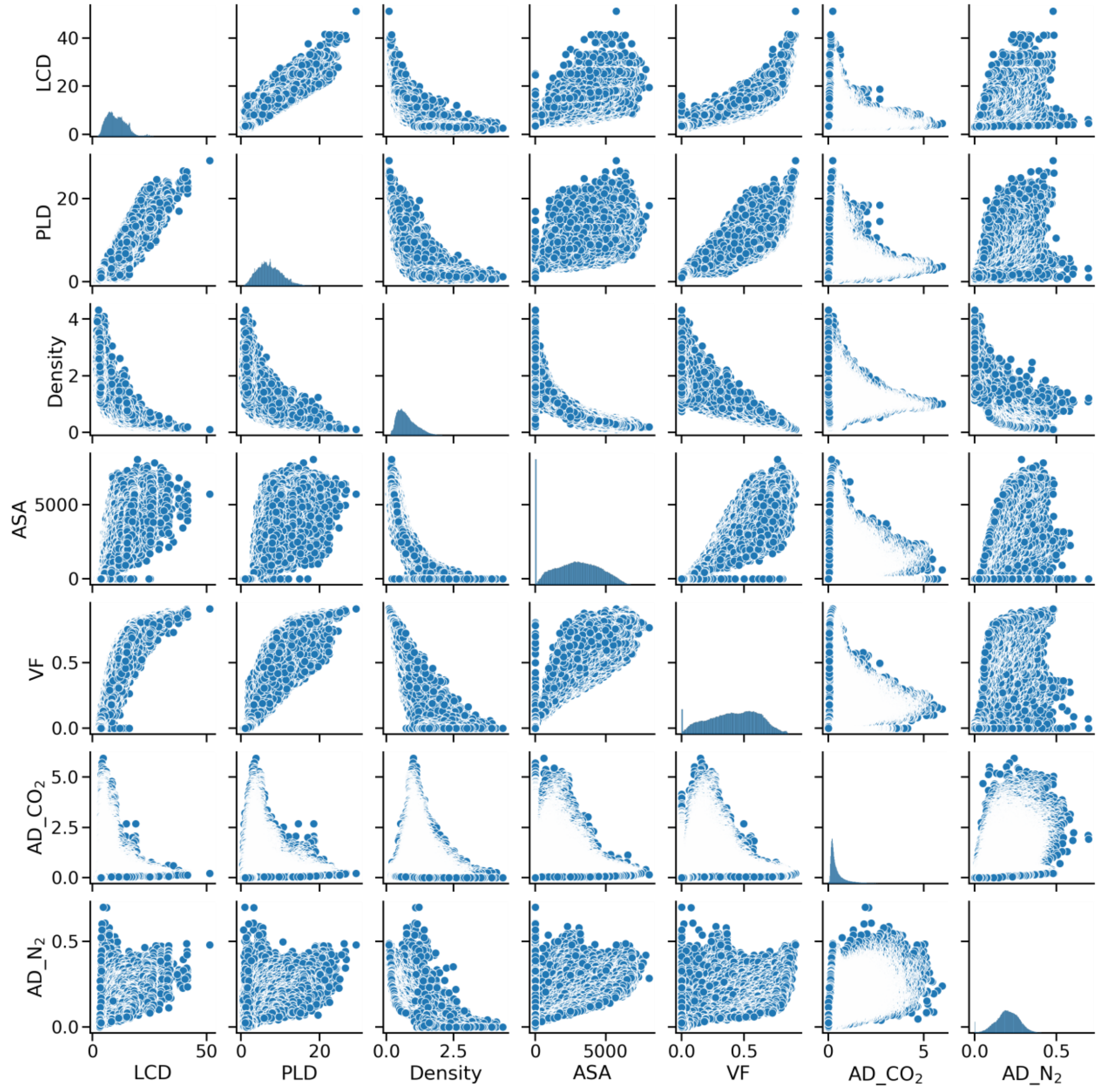

Fig. S7 | Data analysis of hMOF dataset. The relationship between LCD (largest cavity diameter), PLD (pore limiting diameter), Density, ASA (accessible surface area), VF (void fraction), AD_CO_2 (CO_2 adsorption capacity) and AD_N_2 (N_2 adsorption capacity) in hMOF dataset by means of scatter plot.

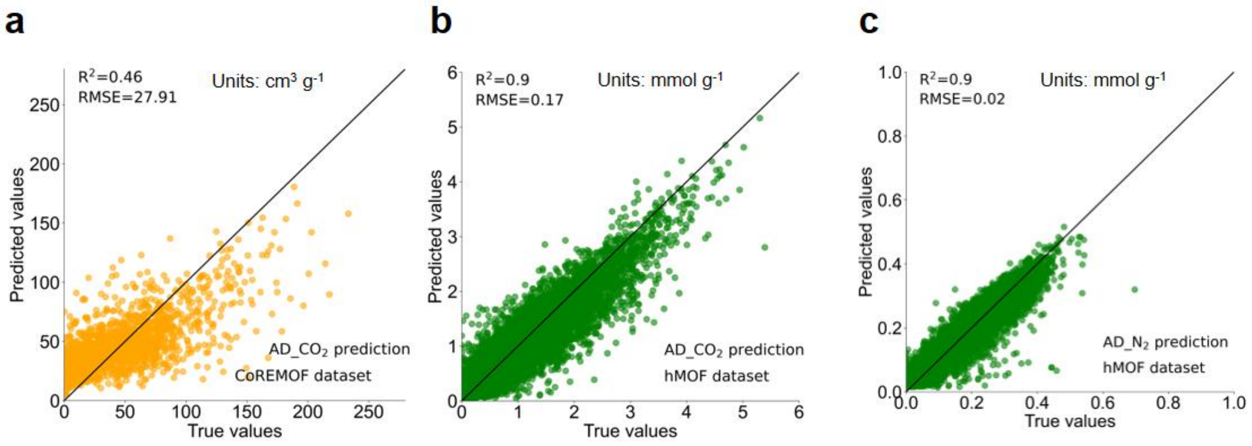

Fig. S8 | Prediction performance of Matformer. a, The correlations between true values and predicted values of AD_CO_2 (CO_2 adsorption capacity) on test set using Matformer on CoREMOF dataset. **b,** The correlations between true values and predicted values of AD_CO_2 (CO_2 adsorption capacity) on test set using Matformer on hMOF dataset. **c,** The correlations between true values and predicted values of AD_N_2 (N_2 adsorption capacity) on test set using Matformer on hMOF dataset.

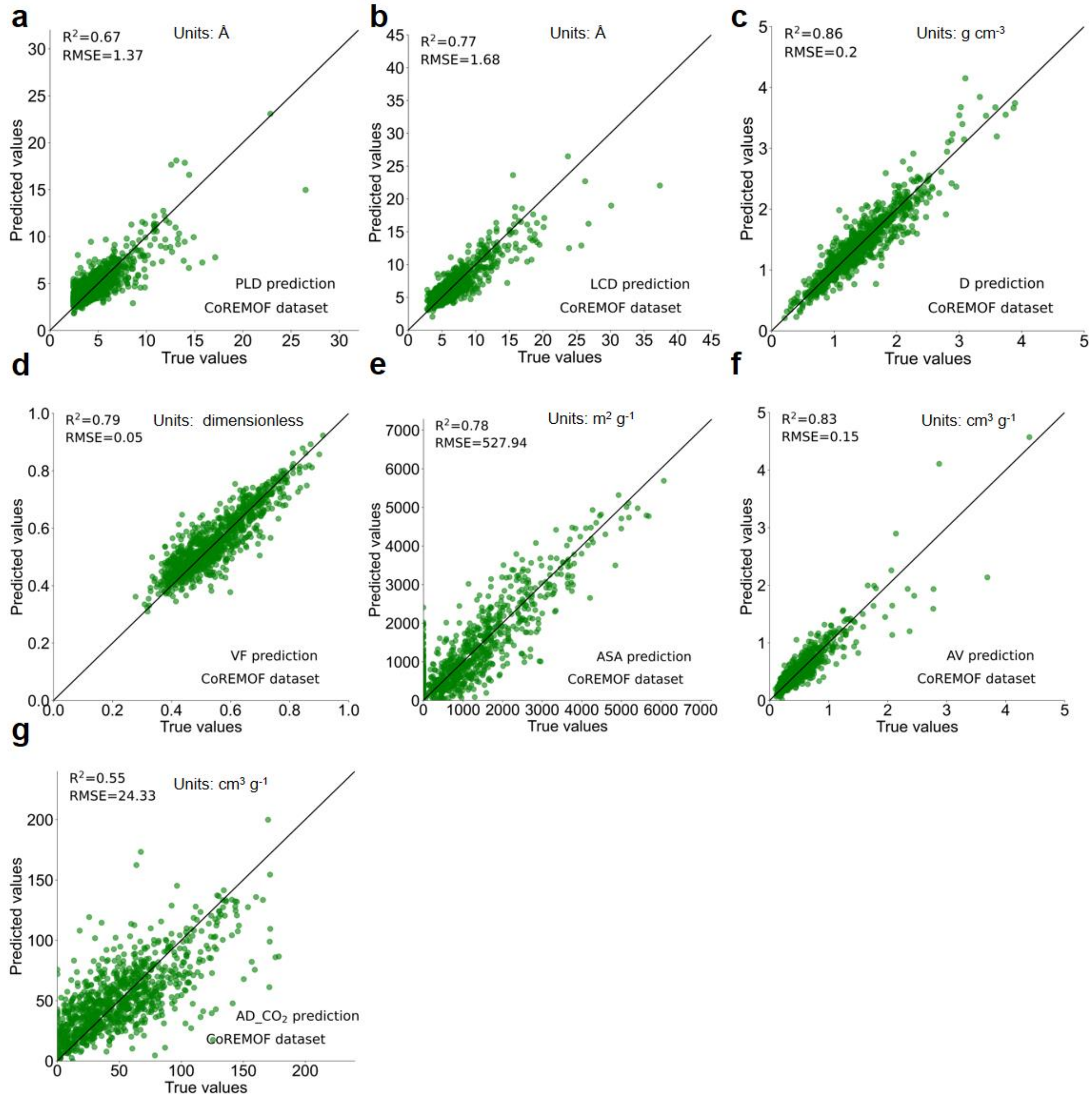

Fig. S9 | Prediction performance of LSTM+KCL on CoREMOF dataset. a, The correlations between true values and predicted values of PLD (pore limiting diameter) on test set using LSTM (Long Short-Term Memory) + KCL (knowledge co-learning) on CoREMOF dataset. **b,** The correlations between true values and predicted values of LCD (largest cavity diameter) on test set using LSTM+KCL on CoREMOF dataset. **c,** The correlations between true values and predicted values of D (density) using LSTM+KCL on CoREMOF dataset. **d,** The correlations between true values and predicted values of VF (void fraction) on test set using LSTM+KCL on CoREMOF dataset. **e,** The correlations between true values and predicted values of ASA (accessible surface area) on test set using LSTM+KCL on CoREMOF dataset. **f,** The correlations between true values and predicted values of AV (accessible volume) on test set using LSTM+KCL on CoREMOF dataset. **g,** The correlations between true values and predicted values of AD_CO_2 (CO_2 adsorption capacity) on test set using LSTM+KCL on CoREMOF dataset.

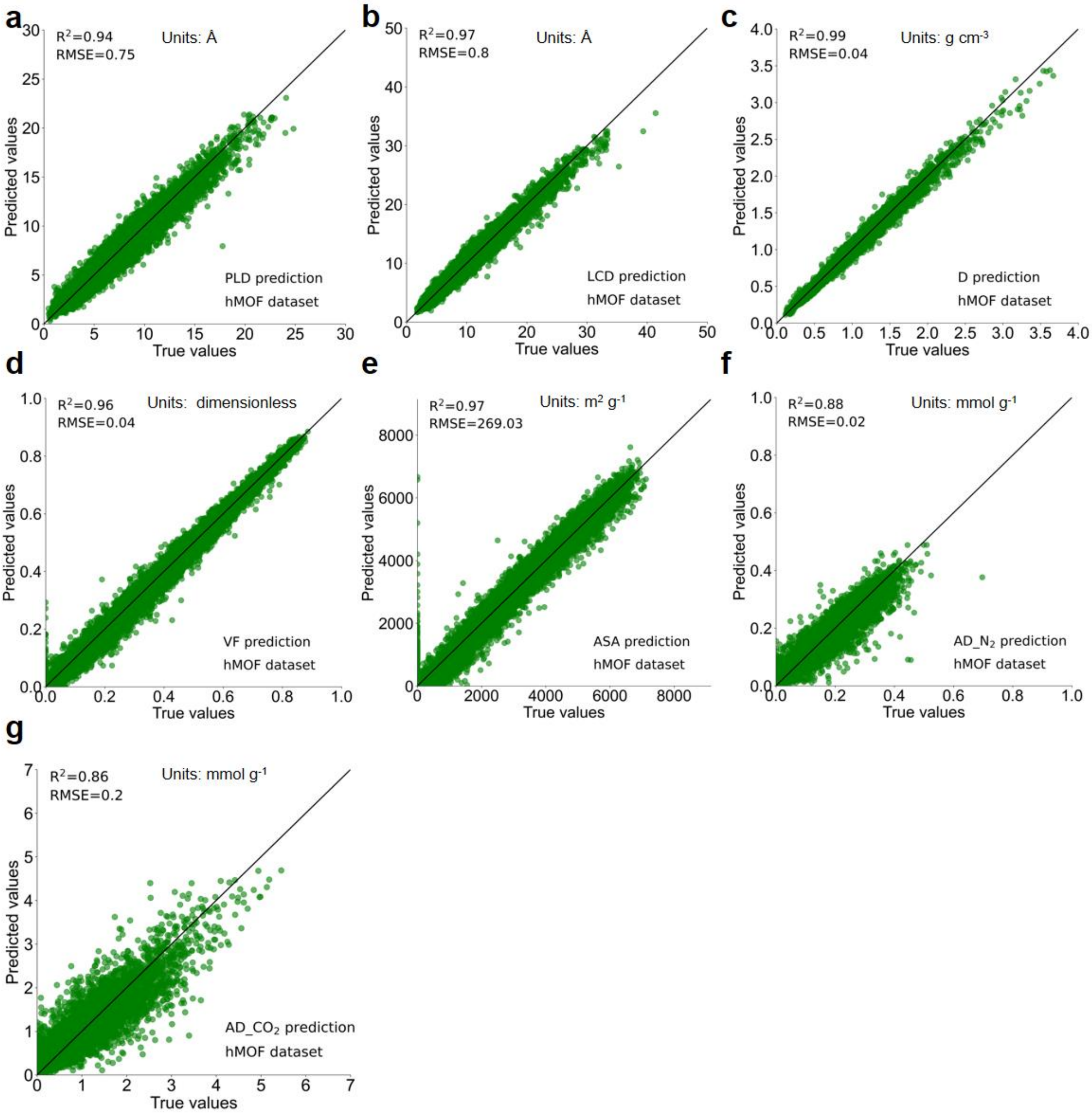

Fig. S10 | Prediction performance of LSTM+KCL on hMOF dataset. a, The correlations between true values and predicted values of PLD (pore limiting diameter) on test set using LSTM (Long Short-Term Memory) + KCL (knowledge co-learning) on hMOF dataset. **b,** The correlations between true values and predicted values of LCD (largest cavity diameter) on test set using LSTM+KCL on hMOF dataset. **c,** The correlations between true values and predicted values of D (density) on test set using LSTM+KCL on hMOF dataset. **d,** The correlations between true values and predicted values of VF (void fraction) on test set using LSTM+KCL on hMOF dataset. **e,** The correlations between true values and predicted values of ASA (accessible surface area) on test set using LSTM+KCL on hMOF dataset. **f,** The correlations between true values and predicted values of AD_N_2 (N_2 adsorption capacity) on test set using LSTM+KCL on hMOF dataset. **g,** The correlations between true values and predicted values of AD_CO_2 (CO_2 adsorption capacity) on test set using LSTM+KCL on hMOF dataset.

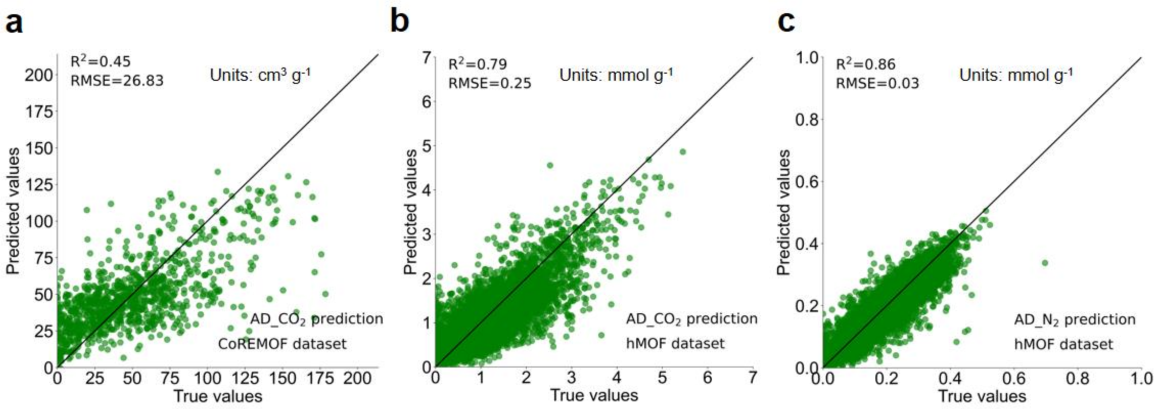

Fig. S11 | Prediction performance of LSTM on CoREMOF and hMOF datasets. a, The correlations between true values and predicted values of AD_CO_2 (CO_2 adsorption capacity) on test set using LSTM (Long Short-Term Memory) on CoREMOF dataset. **b,** The correlations between true values and predicted values of AD_CO_2 (CO_2 adsorption capacity) on test set using LSTM on hMOF dataset. **c,** The correlations between true values and predicted values of AD_N_2 (N_2 adsorption capacity) on test set using LSTM on hMOF dataset.

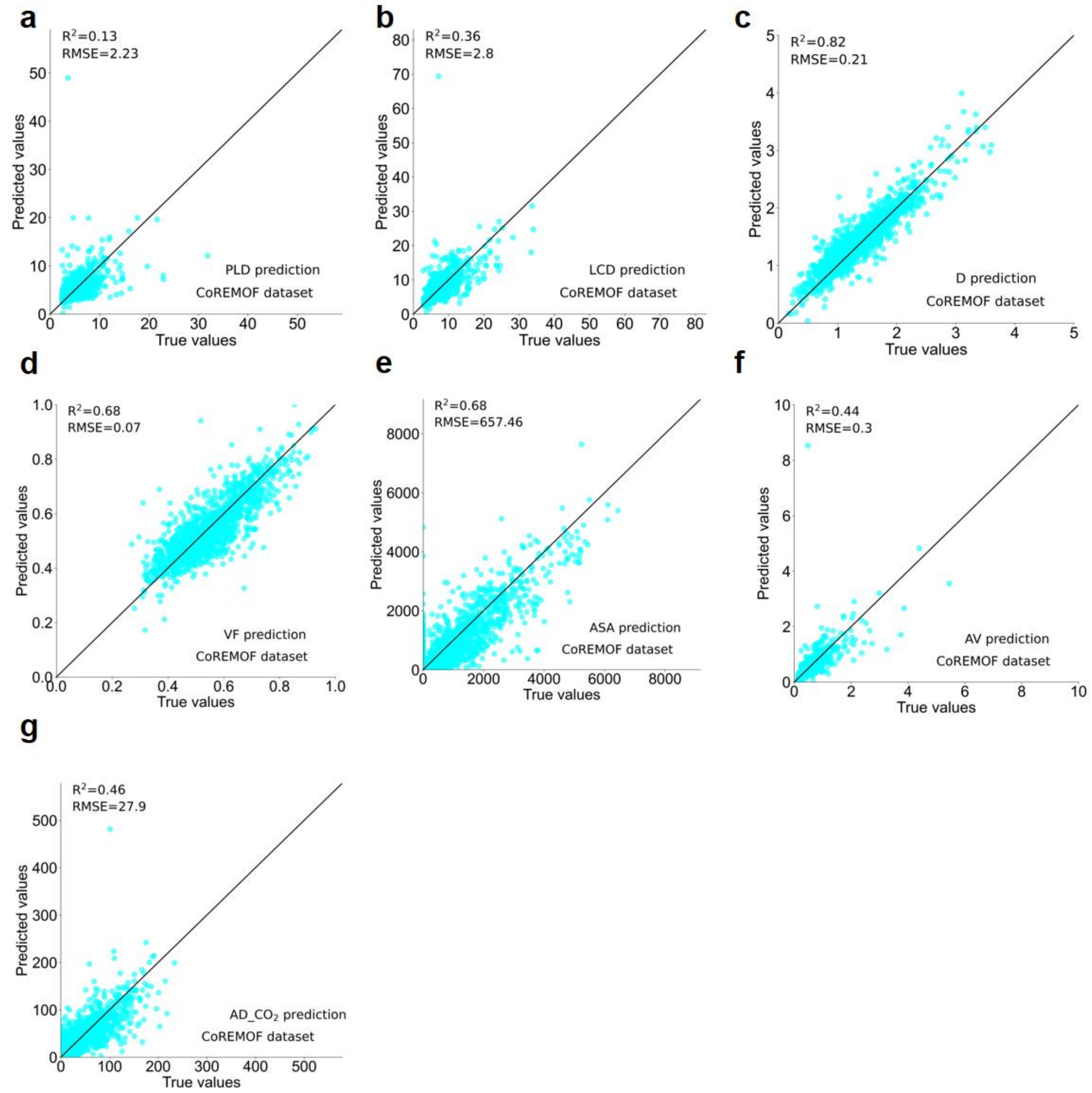

Fig. S12 | Prediction performance of CGCNN+KCL on CoREMOF dataset. a, The correlations between true values and predicted values of PLD (pore limiting diameter) on test set using CGCNN+KCL on CoREMOF dataset. **b,** The correlations between true values and predicted values of LCD (largest cavity diameter) on test set using CGCNN+KCL on CoREMOF dataset. **c,** The correlations between true values and predicted values of D (density) on test set using CGCNN+KCL on CoREMOF dataset. **d,** The correlations between true values and predicted values of VF (void fraction) on test set using CGCNN+KCL on CoREMOF dataset. **e,** The correlations between true values and predicted values of ASA (accessible surface area) on test set using CGCNN+KCL on CoREMOF dataset. **f,** The correlations between true values and predicted values of AV (accessible volume) on test set using CGCNN+KCL on CoREMOF dataset. **g,** The correlations between true values and predicted values of AD_CO_2 (CO_2 adsorption capacity) on test set using CGCNN+KCL on CoREMOF dataset.

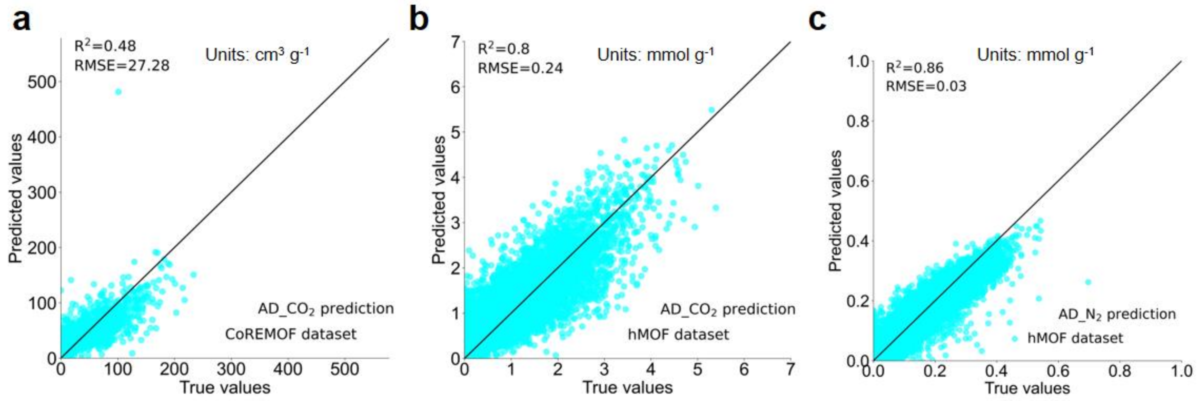

Fig. S13 | Prediction performance of CGCNN on CoREMOF and hMOF datasets. a, The correlations between true values and predicted values of AD_CO_2 (CO_2 adsorption capacity) on test set using CGCNN on CoREMOF dataset. **b,** The correlations between true values and predicted values of AD_CO_2 (CO_2 adsorption capacity) on test set using CGCNN on hMOF dataset. **c,** The correlations between true values and predicted values of AD_N_2 (N_2 adsorption capacity) on test set using CGCNN on hMOF dataset.

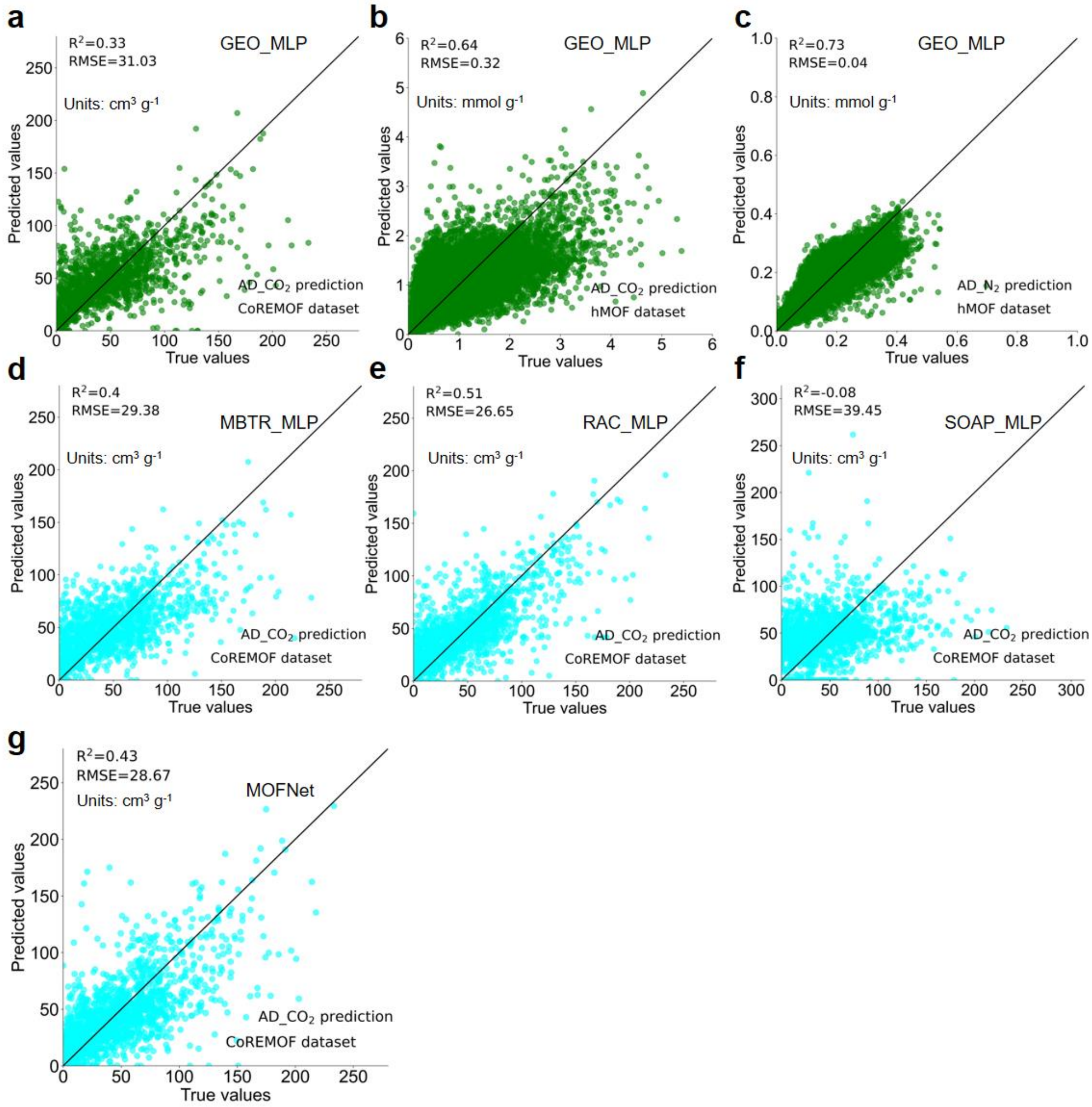

Fig. S14 | Prediction performance of EKDL models and MOFNet on CoREMOF and hMOF dataset. a, The correlations between true values and predicted values of AD_CO_2 (CO_2 adsorption capacity) on test set using GEO_MLP (Geometric structure descriptors multilayer perception models) on CoREMOF dataset. **b,** The correlations between true values and predicted values of AD_CO_2 on test set using GEO_MLP on hMOF dataset. **c,** The correlations between true values and predicted values of AD_N_2 (N_2 adsorption capacity) on test set using GEO_MLP on hMOF dataset. **d,** The correlations between true values and predicted values of AD_CO_2 on test set using MBTR_MLP (Many-body tensor representation descriptors multilayer perception models) on CoREMOF dataset. **e,** The correlations between true values and predicted values of AD_CO_2 on test set using RAC_MLP (Revised autocorrelation functions descriptors multilayer perception models) on CoREMOF dataset. **f,** The correlations between true values and predicted values of AD_CO_2 on test set using SOAP_MLP (Smooth overlap of atomic positions descriptors multilayer perception models) on CoREMOF dataset. **g,** The correlations between true values and predicted values of AD_CO_2 on test set using MOFNet on CoREMOF dataset.

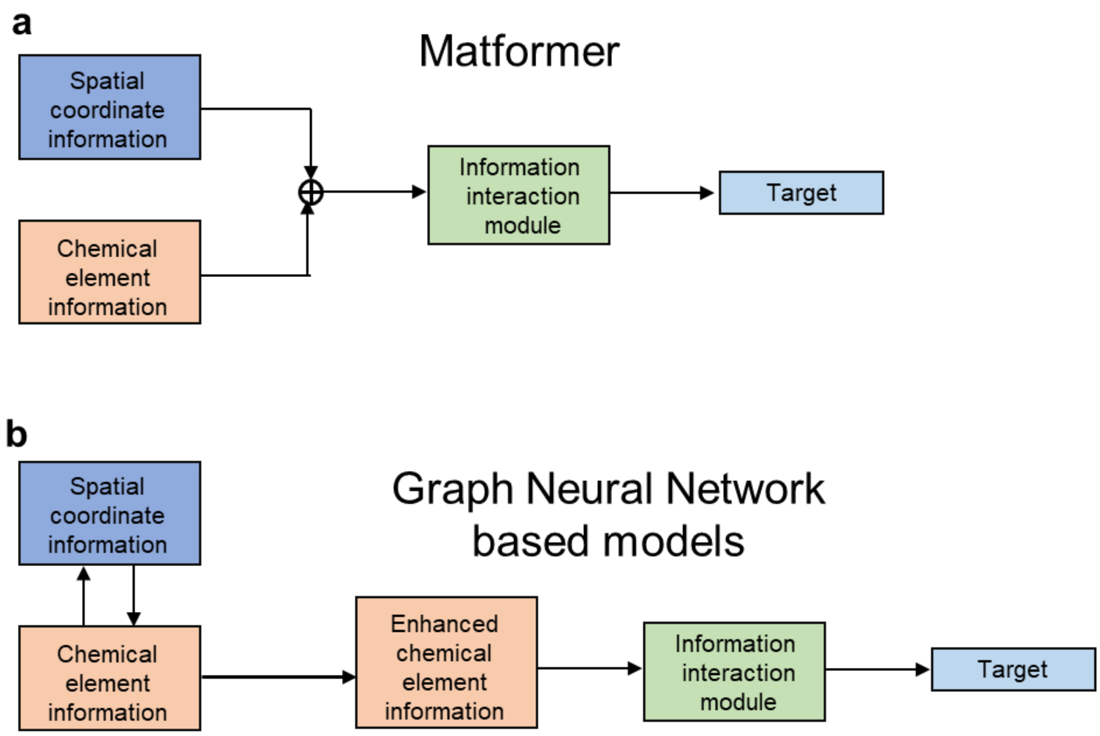

Fig. S15 | The diagrams of information transmission and interaction on Matformer and Graph Neural Network based models. The schematic diagrams show the different spatial coordinate information and chemical element information utilization, transmission and interaction formulas of Matformer (**a**) and Graph Neural Network based models (**b**).

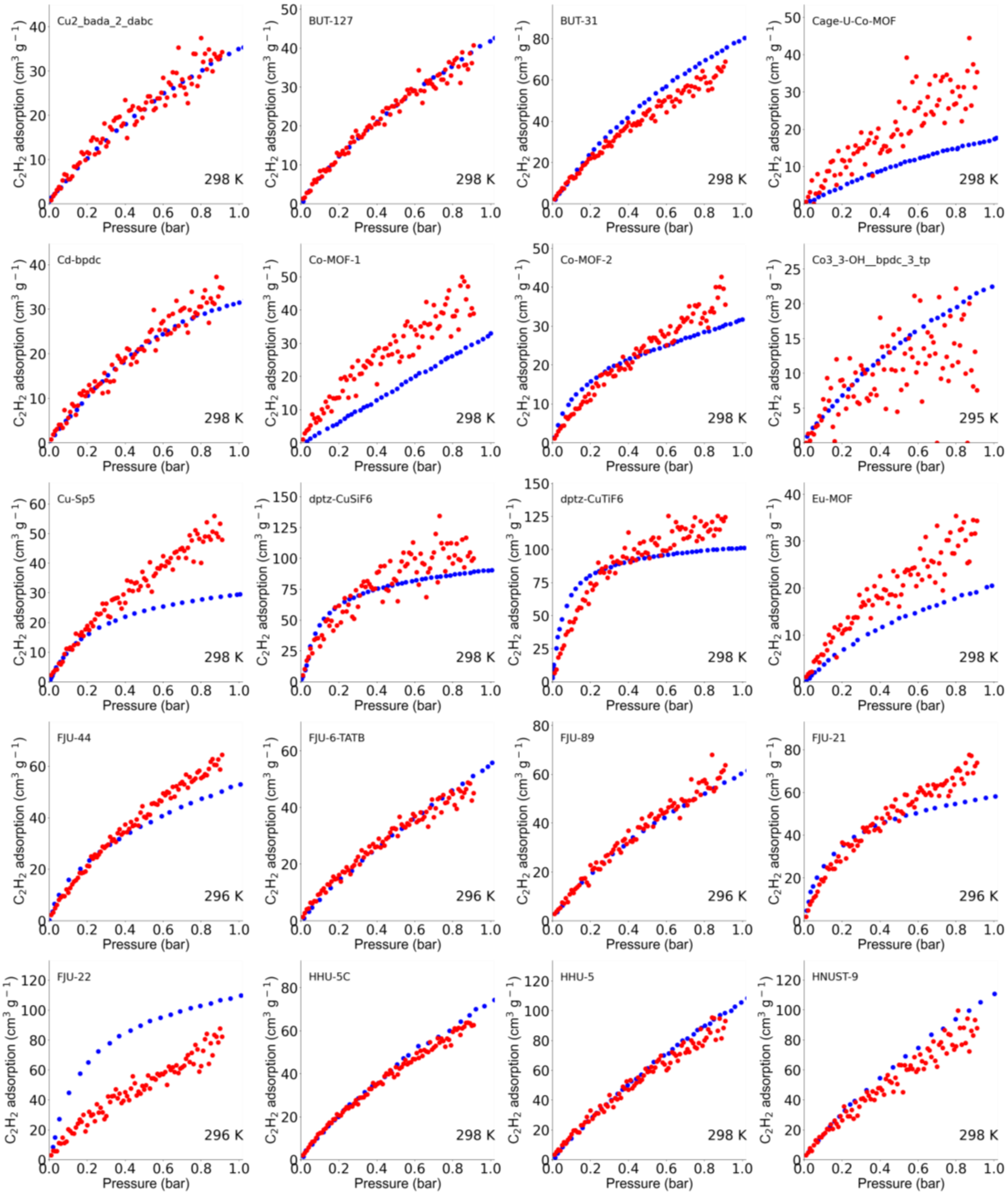

Fig. S16 | Prediction performance of DeepSorption on experimental dataset (EXPMOF-CO_2). The experimental (blue) and predicted (red) adsorption isotherms of MOFs in EXPMOF-CO_2 dataset (including Cu2_bada_2_dabco, BUT-127, BUT-31, Cage-U-Co-MOF, Cd-bpdc, Co-MOF-1, Co-MOF-2, Co3_3-OH__bpdc_3_tpt, Cu-Sp5, dptz-CuSiF$_6$, dptz-CuTiF6, Eu-MOF, FJU-44, FJU-6-TATB, FJU-89, FJU-21, FJU-22, HHU-5C, HHU-5, HNUST-9).

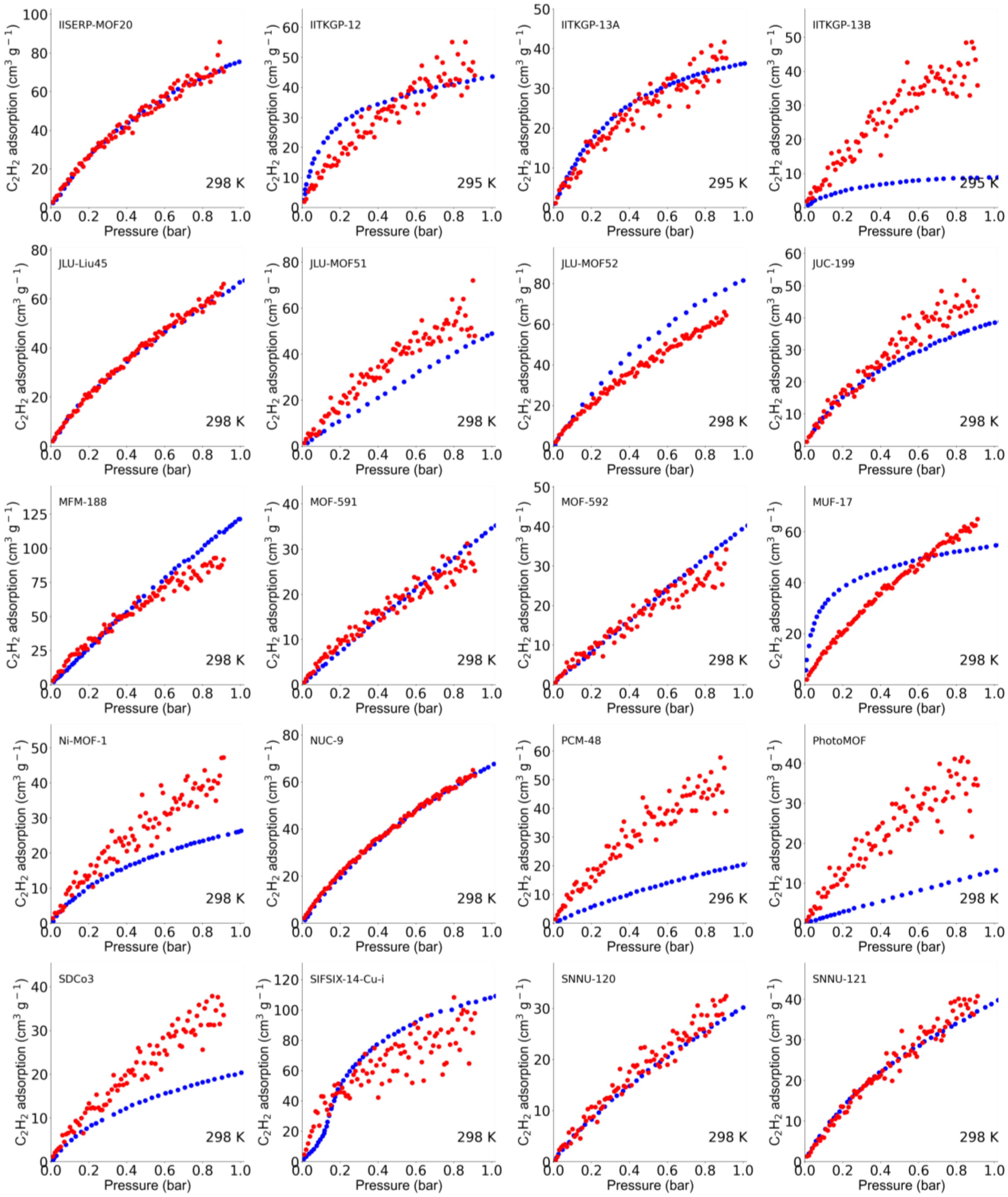

Fig. S17 | Prediction performance of DeepSorption on experimental dataset (EXPMOF-CO_2). The experimental (blue) and predicted (red) adsorption isotherms of MOFs in EXPMOF-CO_2 dataset (including IISERP-MOF20, IITKGP-12, IITKGP-13A, IITKGP-13B, JLU-Liu45, JLU-MOF51, JLU-MOF52, JUC-199, MFM-188, MOF-591, MOF-592, MUF-17, Ni-MOF-1, NUC-9, PCM-48, PhotoMOF, SDCo3, SIFSIX-14-Cu-i, SNNU-120, SNNU-121).

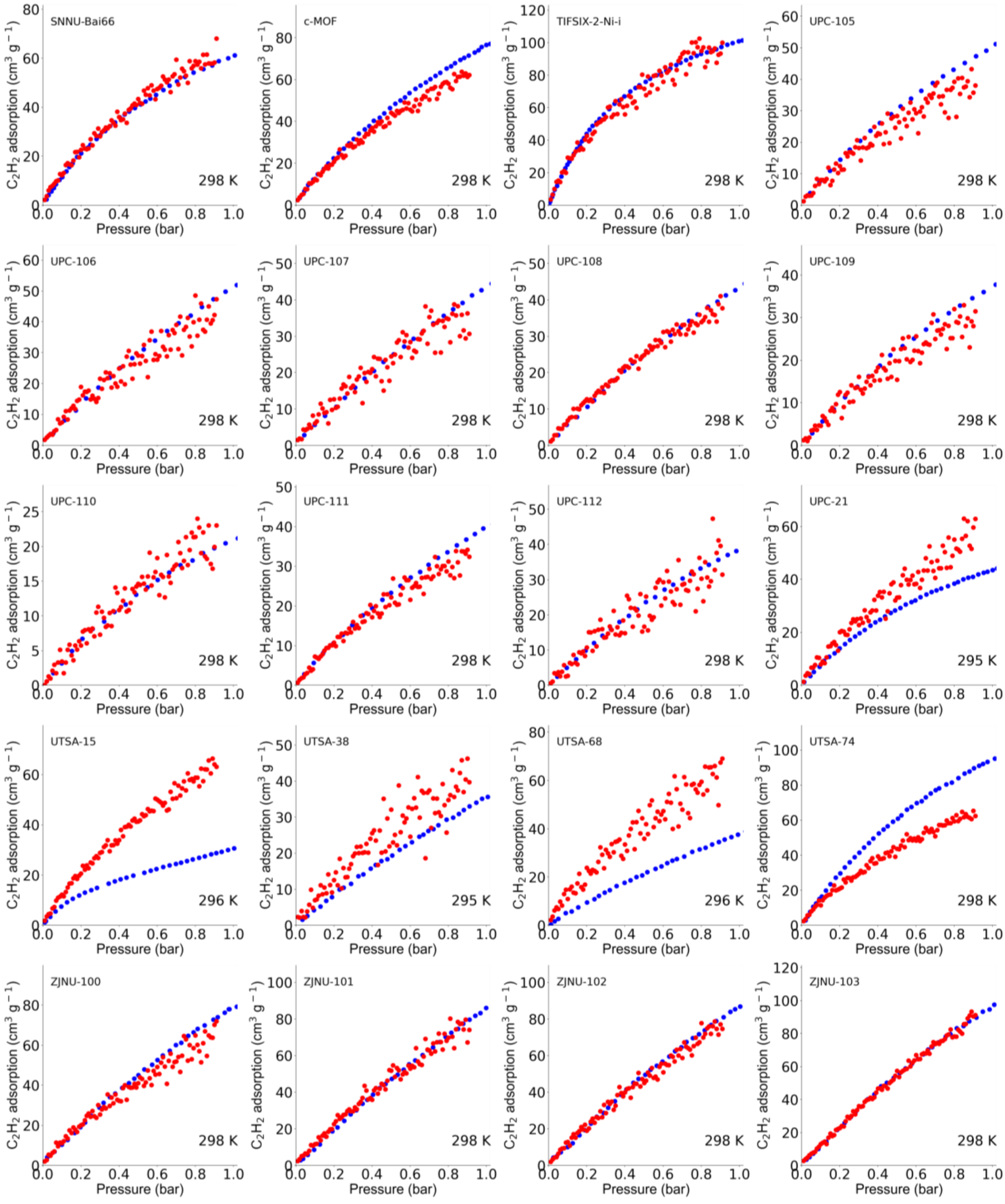

Fig. S18 | Prediction performance of DeepSorption on experimental dataset (EXPMOF-CO_2). The experimental (blue) and predicted (red) adsorption isotherms of MOFs in EXPMOF-CO_2 dataset (including SNNU-Bai66, soc-MOF, TIFSIX-2-Ni-i, UPC-105, UPC-106, UPC-107, UPC-108, UPC-109, UPC-110, UPC-111, UPC-112, UPC-21, UTSA-15, UTSA-38, UTSA-68, UTSA-74, ZJNU-100, ZJNU-101, ZJNU-102, ZJNU-103).

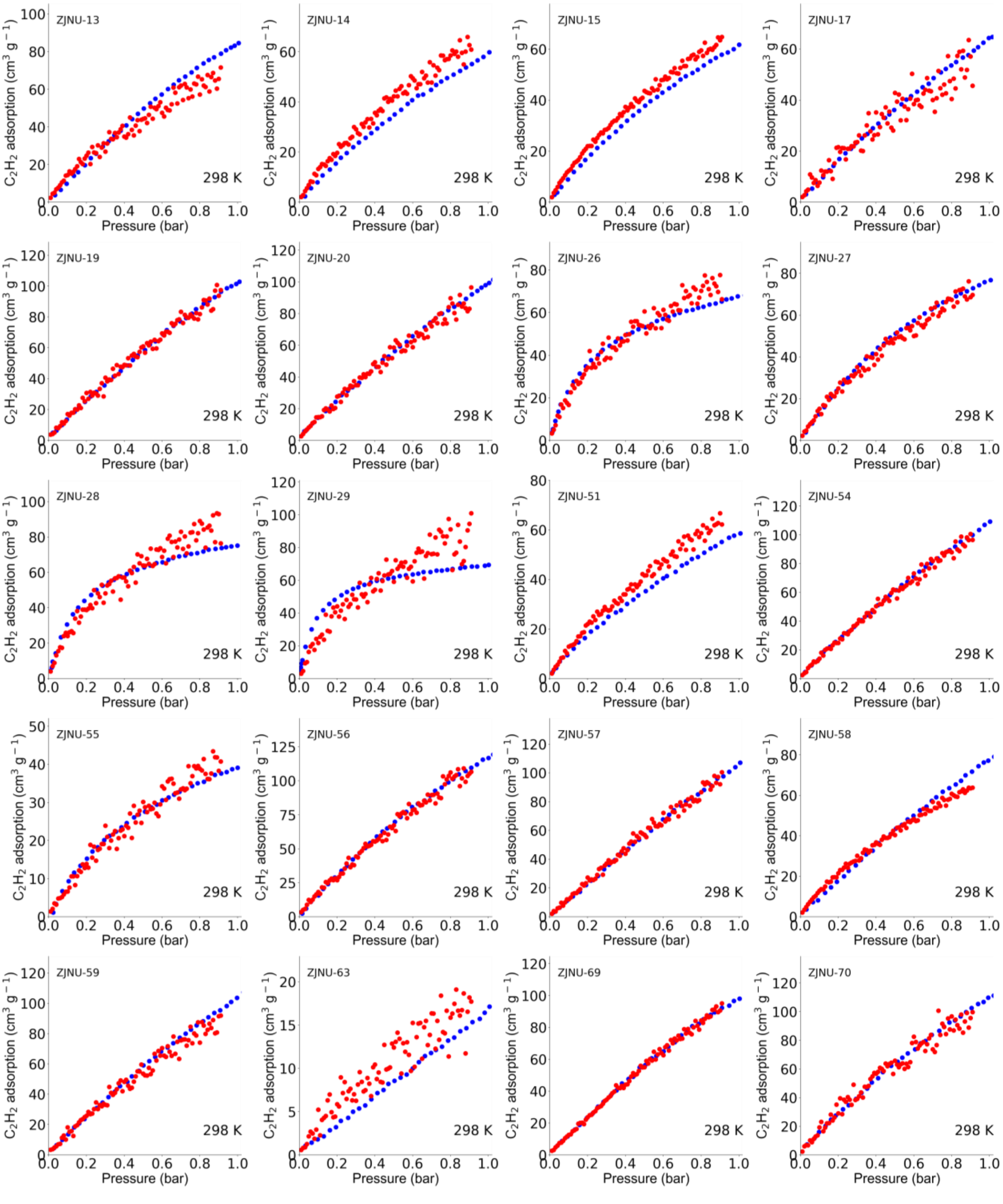

Fig. S19 | Prediction performance of DeepSorption on experimental dataset (EXPMOF-CO_2). The experimental (blue) and predicted (red) adsorption isotherms of MOFs in EXPMOF-CO_2 dataset (including ZJNU-13, ZJNU-14, ZJNU-15, ZJNU-17, ZJNU-19, ZJNU-20, ZJNU-26, ZJNU-27, ZJNU-28, ZJNU-29, ZJNU-51, ZJNU-54, ZJNU-55, ZJNU-56, ZJNU-57, ZJNU-58, ZJNU-59, ZJNU-63, ZJNU-69, ZJNU-70).

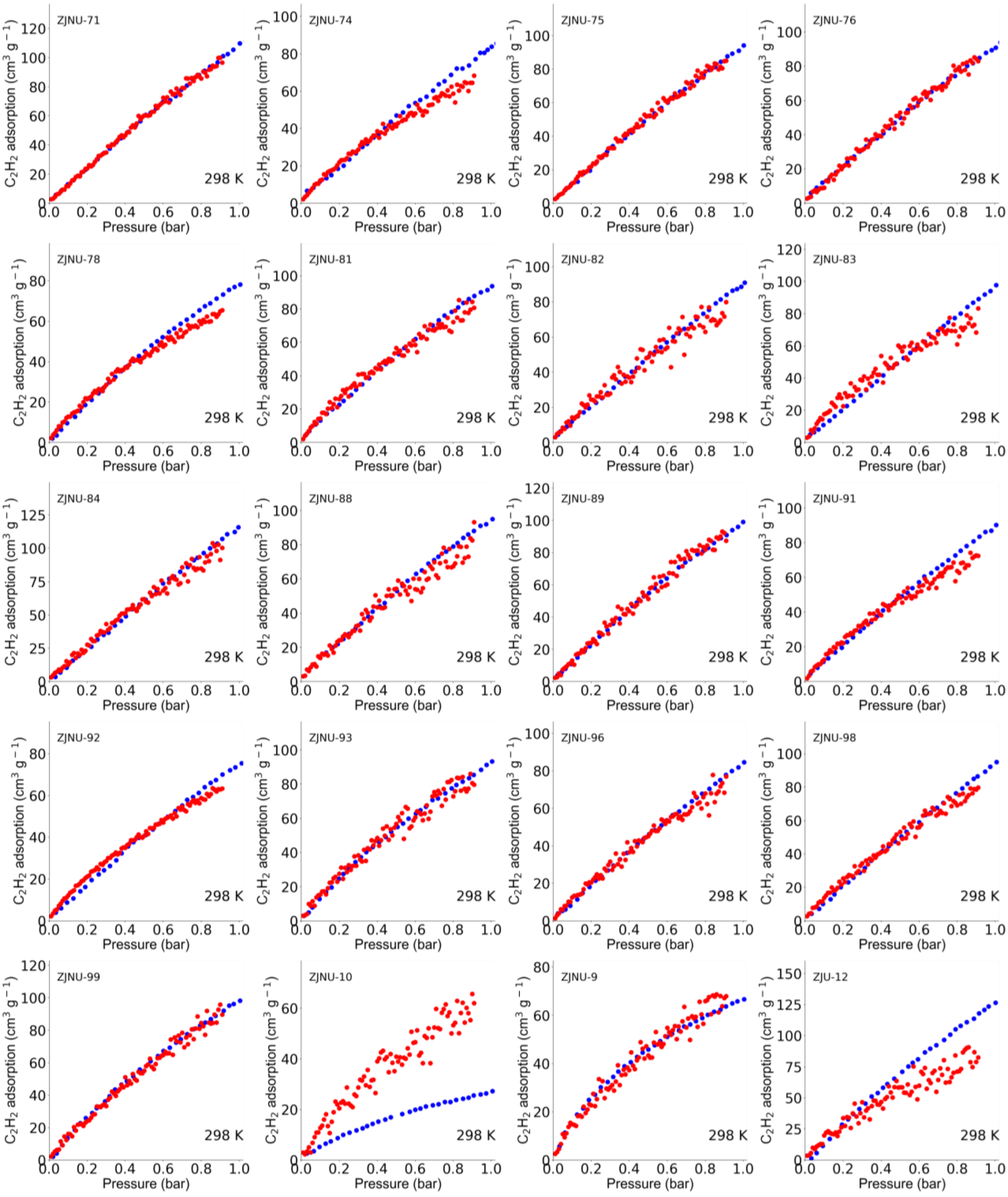

Fig. S20 | Prediction performance of DeepSorption on experimental dataset (EXPMOF-CO_2). The experimental (blue) and predicted (red) adsorption isotherms of MOFs in EXPMOF-CO_2 dataset (including ZJNU-71, ZJNU-74, ZJNU-75, ZJNU-76, ZJNU-78, ZJNU-81, ZJNU-82, ZJNU-83, ZJNU-84, ZJNU-88, ZJNU-89, ZJNU-91, ZJNU-92, ZJNU-93, ZJNU-96, ZJNU-98, ZJNU-99, ZJNU-10, ZJNU-9, ZJU-12).

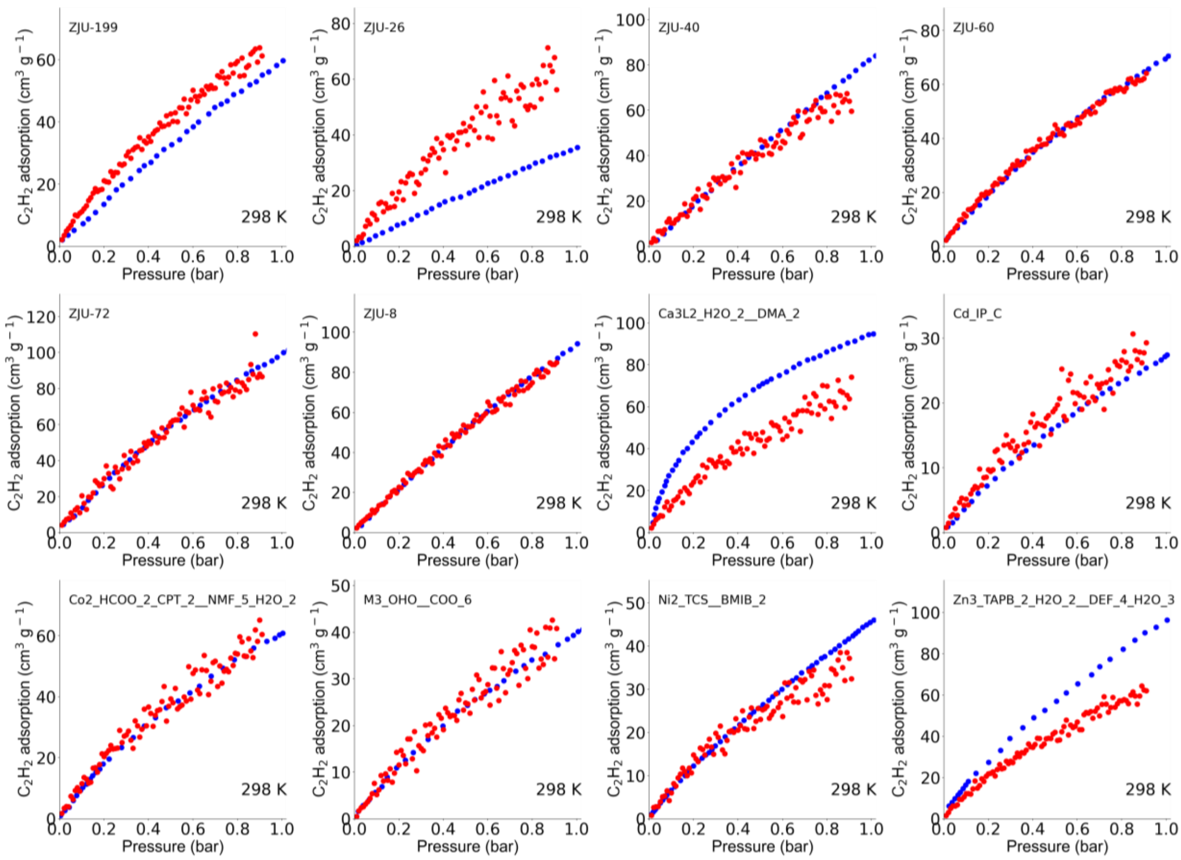

Fig. S21 | Prediction performance of DeepSorption on experimental dataset (EXPMOF-CO_2). The experimental (blue) and predicted (red) adsorption isotherms of MOFs in EXPMOF-CO_2 dataset (including ZJU-199, ZJU-26, ZJU-40, ZJU-60, ZJU-72, ZJU-8, Ca3L2_H2O_2__DMA_2, Cd_IP_Cl_n, Co2_HCOO_2_CPT_2__NMF_5_H_2O_2, M3_OHO__COO_6, Ni2_TCS__BMIB_2, Zn3_TAPB_2_H2O_2__DEF_4_H2O_3).

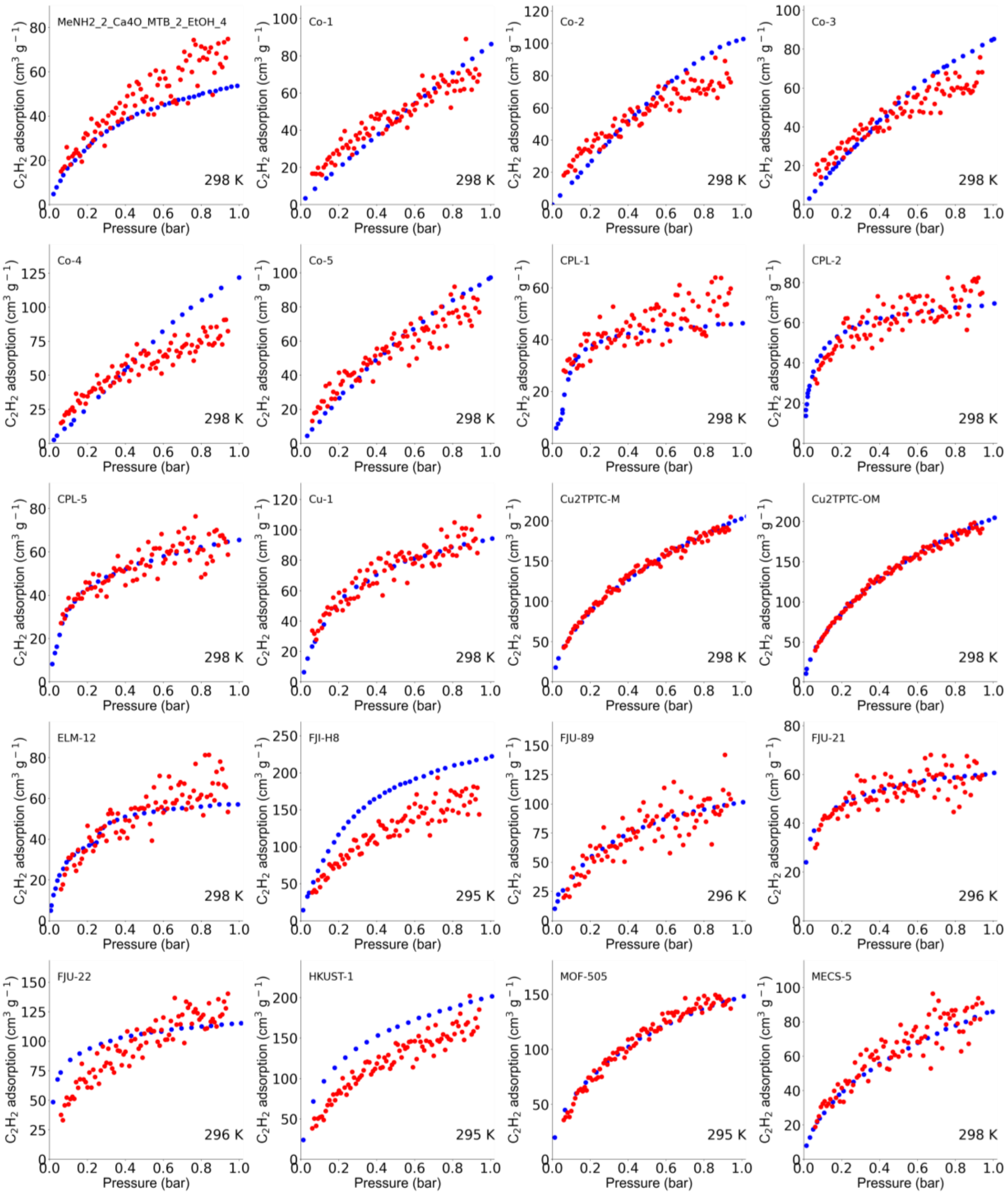

Fig. S22 | Prediction performance of DeepSorption on experimental dataset (EXPMOF-C_2H_2). The experimental (blue) and predicted (red) adsorption isotherms of MOFs in EXPMOF-C_2H_2 dataset (including MeNH2_2_Ca4O_MTB_2_EtOH_4, Co-1, Co-2, Co-3, Co-4, Co-5, CPL-1, CPL-2, CPL-5, Cu-1, Cu2TPTC-Me, Cu2TPTC-OMe, ELM-12, FJI-H8, FJU-89, FJU-21, FJU-22, HKUST-1, MOF-505, MECS-5).

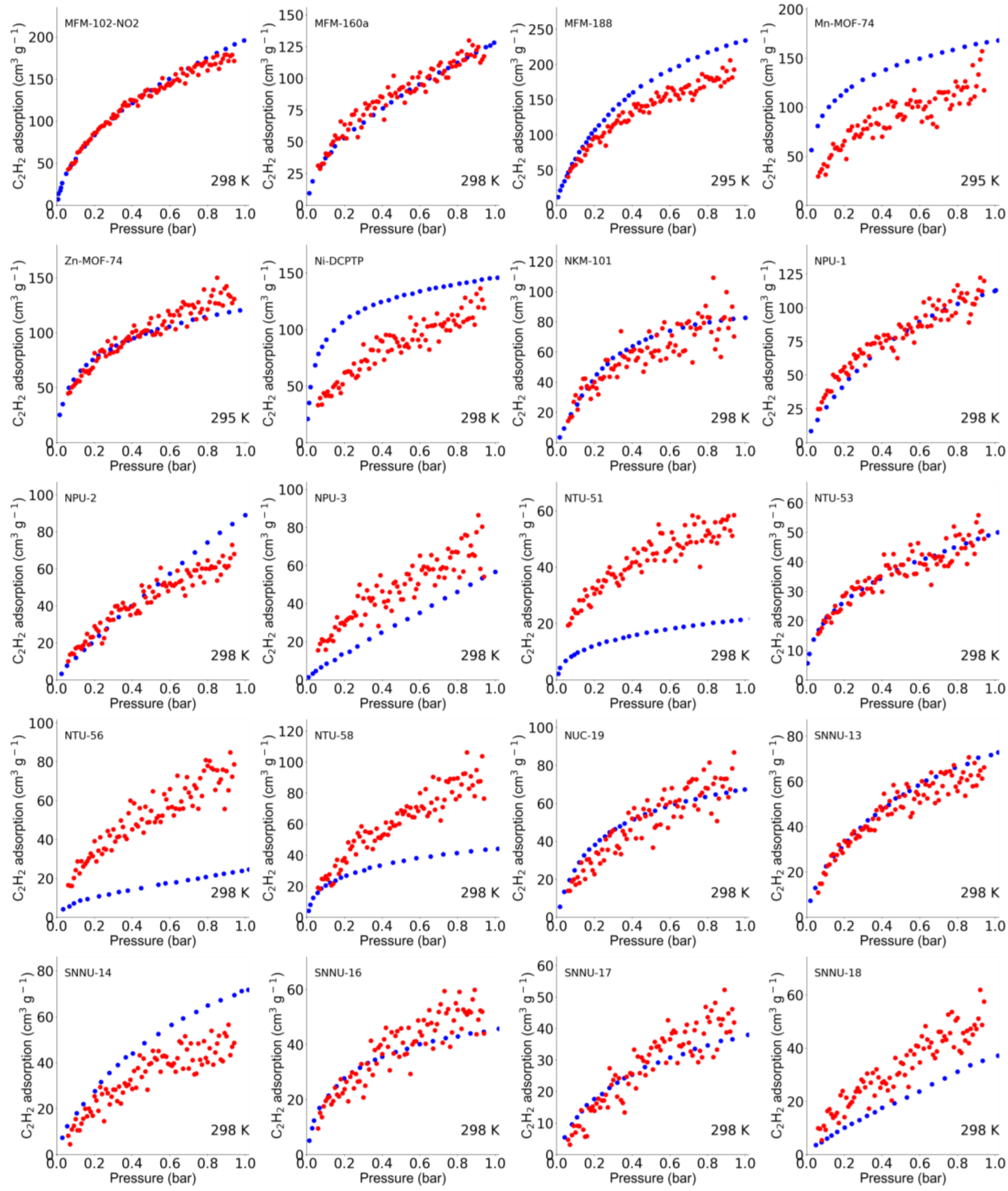

Fig. S23 | Prediction performance of DeepSorption on experimental dataset (EXPMOF-C_2H_2). The experimental (blue) and predicted (red) adsorption isotherms of MOFs in EXPMOF-C_2H_2 dataset (including MFM-102-NO_2, MFM-160a, MFM-188, Mn-MOF-74, Zn-MOF-74, Ni-DCPTP, NKM-101, NPU-1, NPU-2, NPU-3, NTU-51, NTU-53, NTU-56, NTU-58, NUC-19, SNNU-13, SNNU-14, SNNU-16, SNNU-17, SNNU-18).

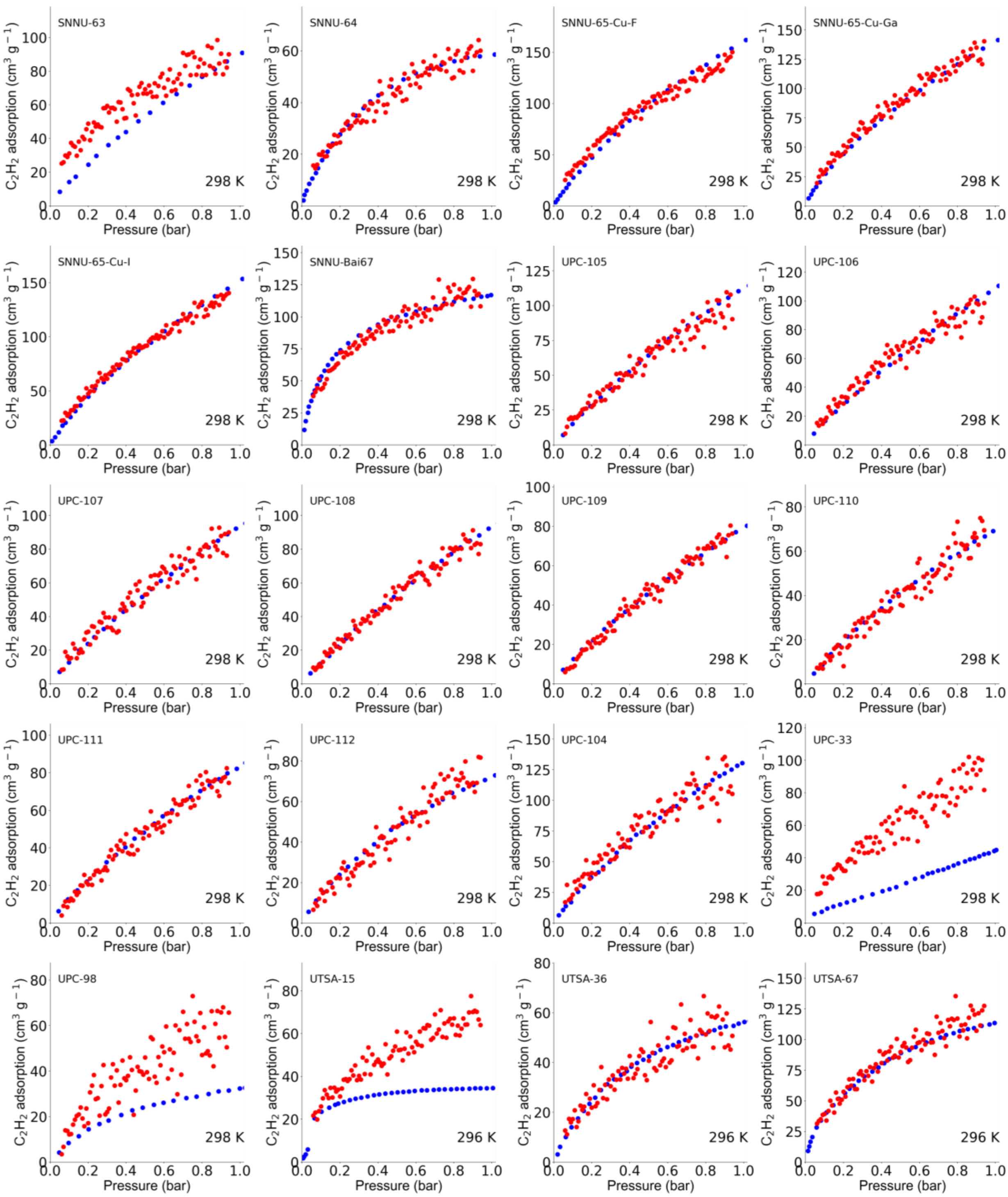

Fig. S24 | Prediction performance of DeepSorption on experimental dataset (EXPMOF-C_2H_2). The experimental (blue) and predicted (red) adsorption isotherms of MOFs in EXPMOF-C_2H_2 dataset (including SNNU-63, SNNU-64, SNNU-65-Cu-Fe, SNNU-65-Cu-Ga, SNNU-65-Cu-In, SNNU-Bai67, UPC-105, UPC-106, UPC-107, UPC-108, UPC-109, UPC-110, UPC-111, UPC-112, UPC-104, UPC-33, UPC-98, UTSA-15, UTSA-36, UTSA-67).

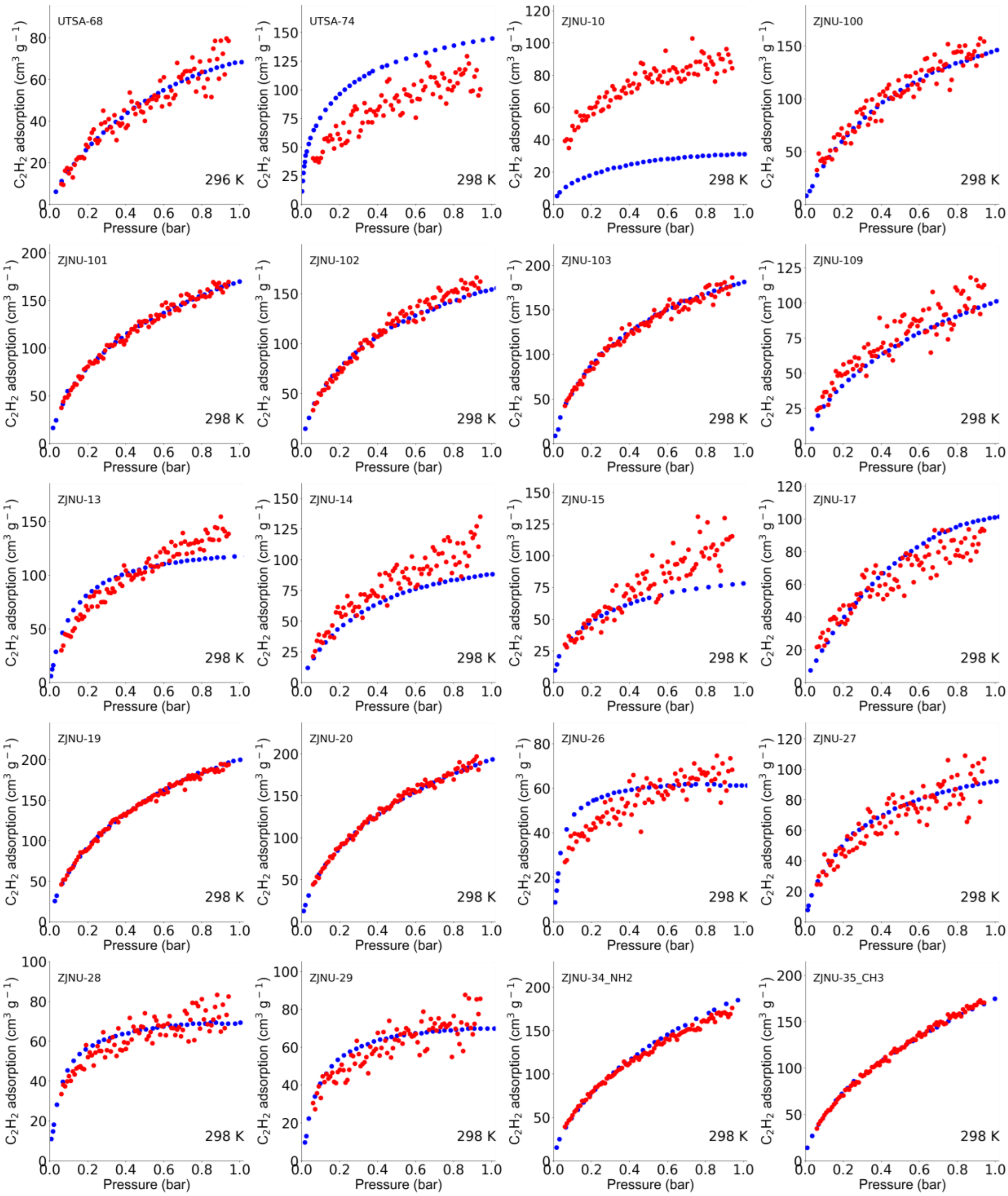

Fig. S25 | Prediction performance of DeepSorption on experimental dataset (EXPMOF-C_2H_2). The experimental (blue) and predicted (red) adsorption isotherms of MOFs in EXPMOF-C_2H_2 dataset (including UTSA-68, UTSA-74, ZJNU-10, ZJNU-100, ZJNU-101, ZJNU-102, ZJNU-103, ZJNU-109, ZJNU-13, ZJNU-14, ZJNU-15, ZJNU-17, ZJNU-19, ZJNU-20, ZJNU-26, ZJNU-27, ZJNU-28, ZJNU-29, ZJNU-34_NH2, ZJNU-35_CH_3).

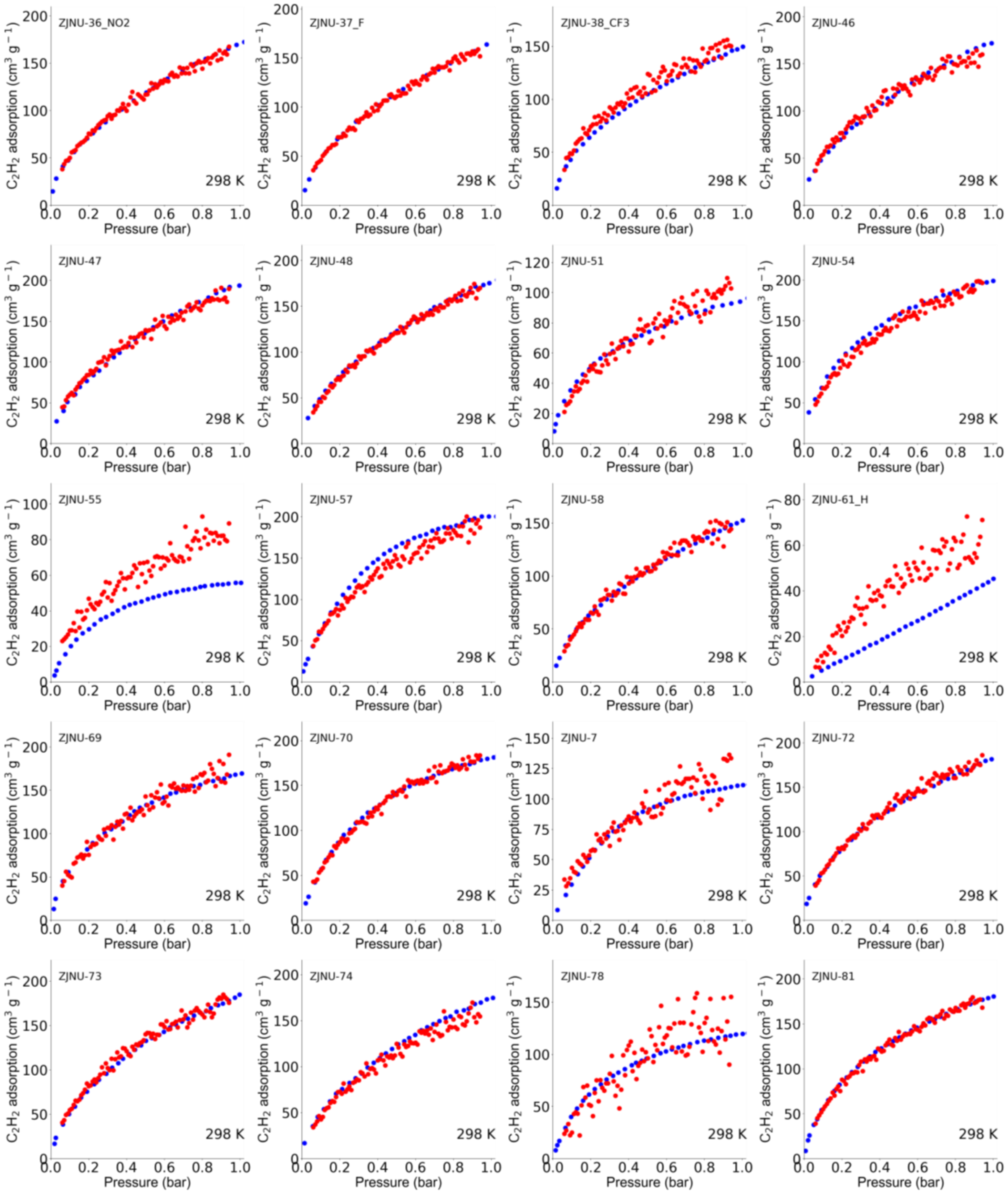

Fig. S26 | Prediction performance of DeepSorption on experimental dataset (EXPMOF-C_2H_2). The experimental (blue) and predicted (red) adsorption isotherms of MOFs in EXPMOF-C_2H_2 dataset (including ZJNU-36_NO2, ZJNU-37_F, ZJNU-38_CF3, ZJNU-46, ZJNU-47, ZJNU-48, ZJNU-51, ZJNU-54, ZJNU-55, ZJNU-57, ZJNU-58, ZJNU-61_Ho, ZJNU-69, ZJNU-70, ZJNU-7, ZJNU-72, ZJNU-73, ZJNU-74, ZJNU-78, ZJNU-81).

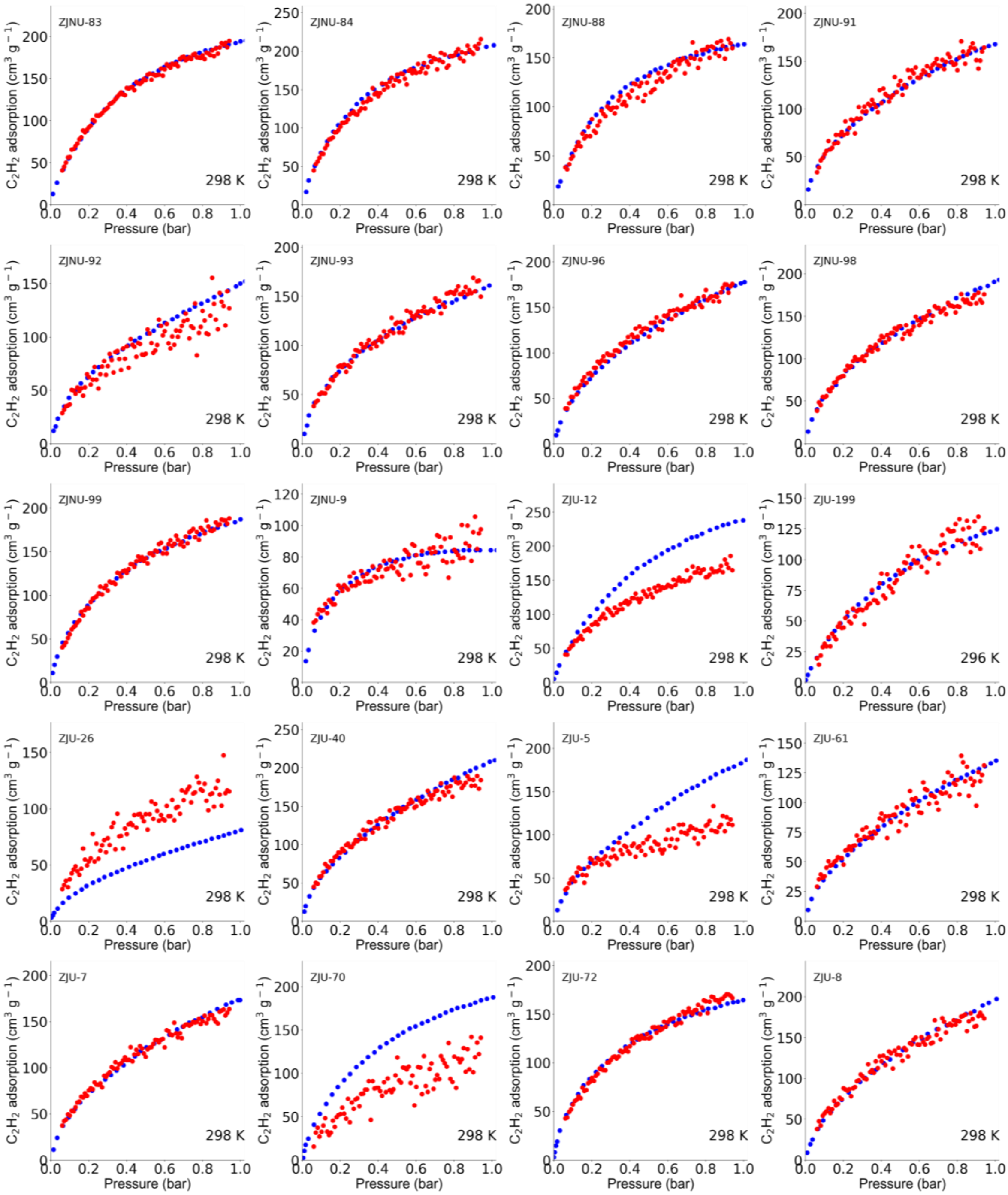

Fig. S27 | Prediction performance of DeepSorption on experimental dataset (EXPMOF-C_2H_2). The experimental (blue) and predicted (red) adsorption isotherms of MOFs in EXPMOF-C_2H_2 dataset (including ZJNU-83, ZJNU-84, ZJNU-88, ZJNU-91, ZJNU-92, ZJNU-93, ZJNU-96, ZJNU-98, ZJNU-99, ZJNU-9, ZJU-12, ZJU-199, ZJU-26, ZJU-40, ZJU-5, ZJU-61, ZJU-7, ZJU-70, ZJU-72, ZJU-8).

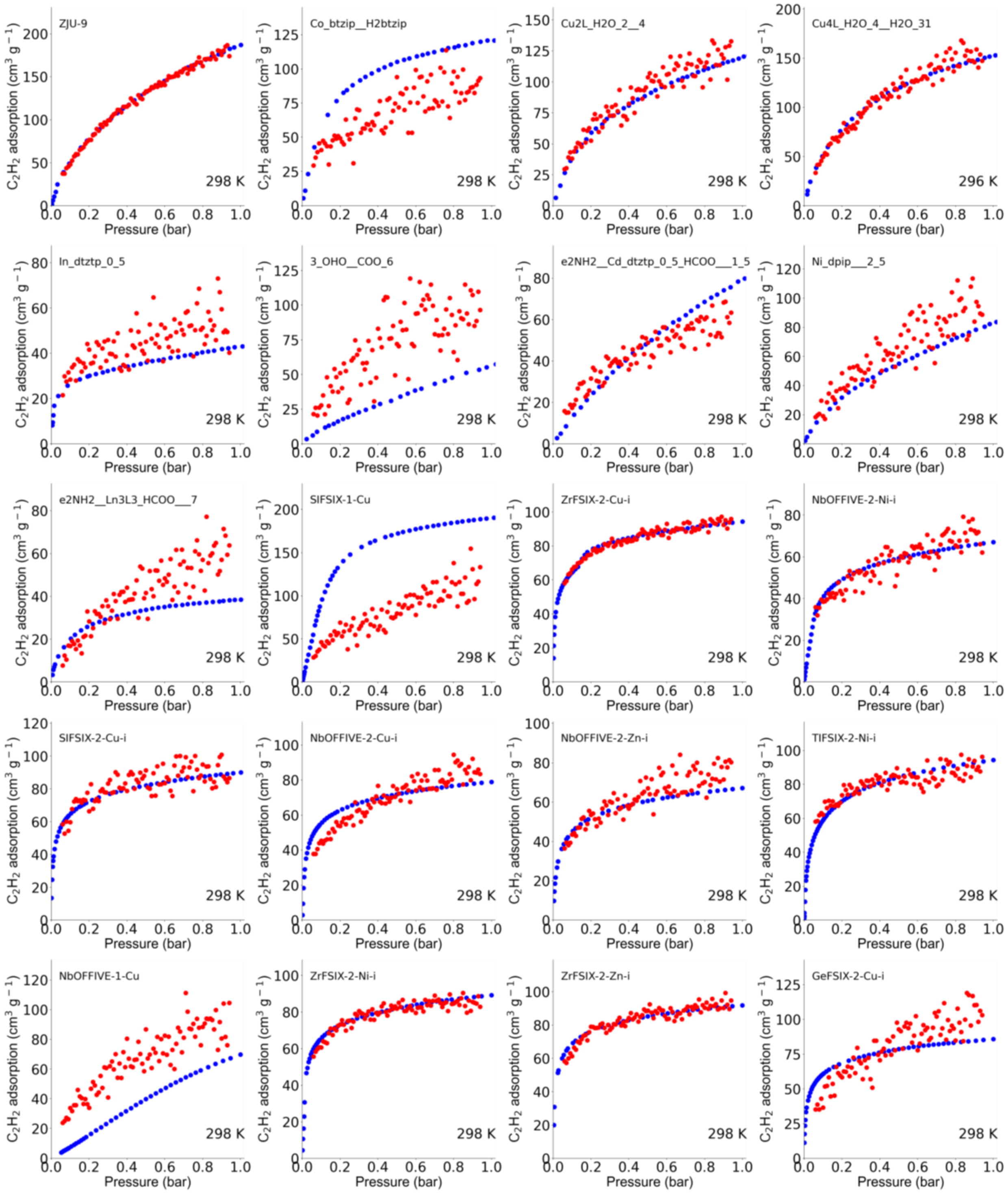

Fig. S28 | Prediction performance of DeepSorption on experimental dataset (EXPMOF-C_2H_2). The experimental (blue) and predicted (red) adsorption isotherms of MOFs in EXPMOF-C_2H_2 dataset (including ZJU-9, Co_btzip__H2btzip, Cu2L_H_2O_2__4, Cu_4L_H_2O_4__H_2O_31, In_dtztp_0_5, M3_OHO__COO_6, Me_2NH2__Cd_dtztp_0_5_HCOO___1_5, Ni_dpip___2_5, Me_2NH_2__Ln_3L_3_HCOO___7, SIFSIX-1-Cu, ZrFSIX-2-Cu-i, NbOFFIVE-2-Ni-i, SIFSIX-2-Cu-i, NbOFFIVE-2-Cu-i, NbOFFIVE-2-Zn-i, TIFSIX-2-Ni-i, NbOFFIVE-1-Cu, ZrFSIX-2-Ni-i, ZrFSIX-2-Zn-i, GeFSIX-2-Cu-i).

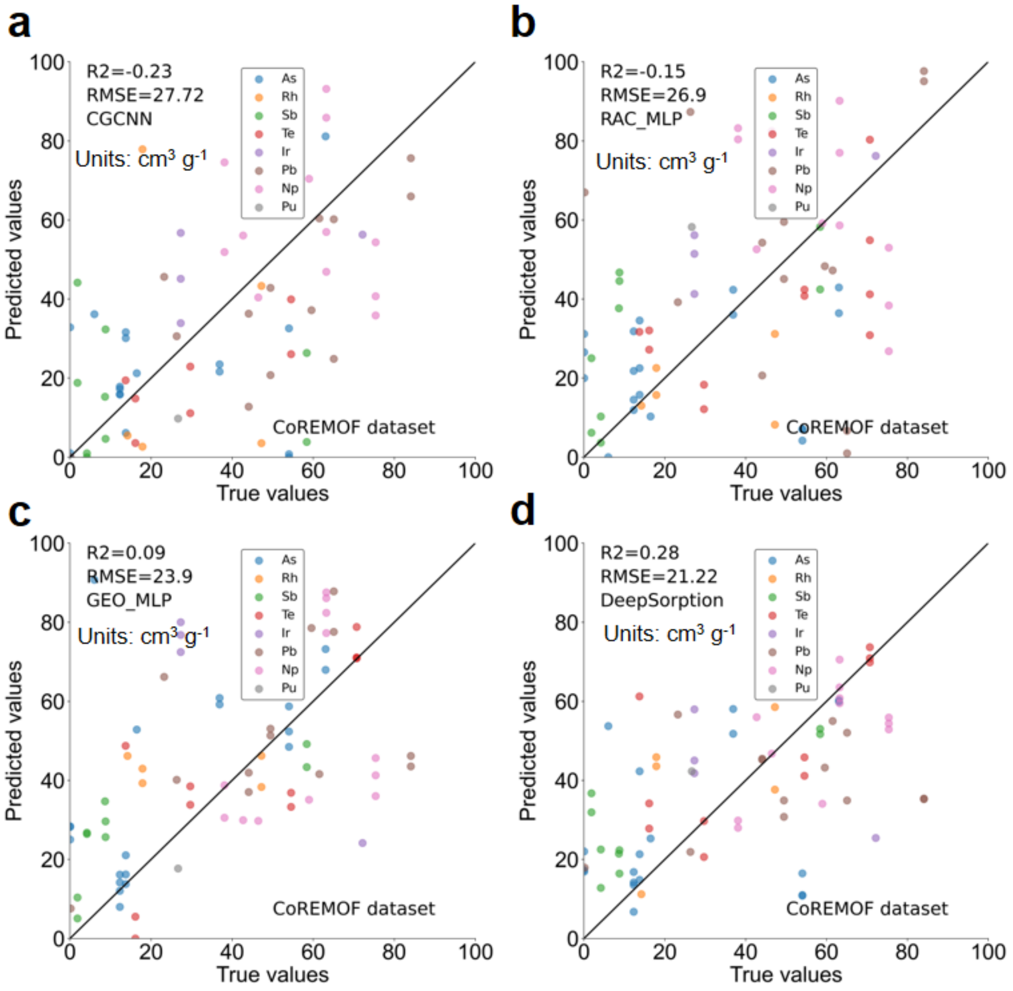

Fig. S29 | Adsorption capacity prediction performance of materials with rare elements in CoREMOF dataset. a, The correlations between true values and predicted values of CO_2 adsorption capacity of materials with rare elements (As, Rh, Sb, Te, Ir, Pb, Np and Pu) on test set with 10 different random divisions using CGCNN (**a**), RAC_MLP (**b**), GEO_MLP (**c**) and DeepSorption (MatFormer+KCL) (**d**) on CoREMOF dataset.

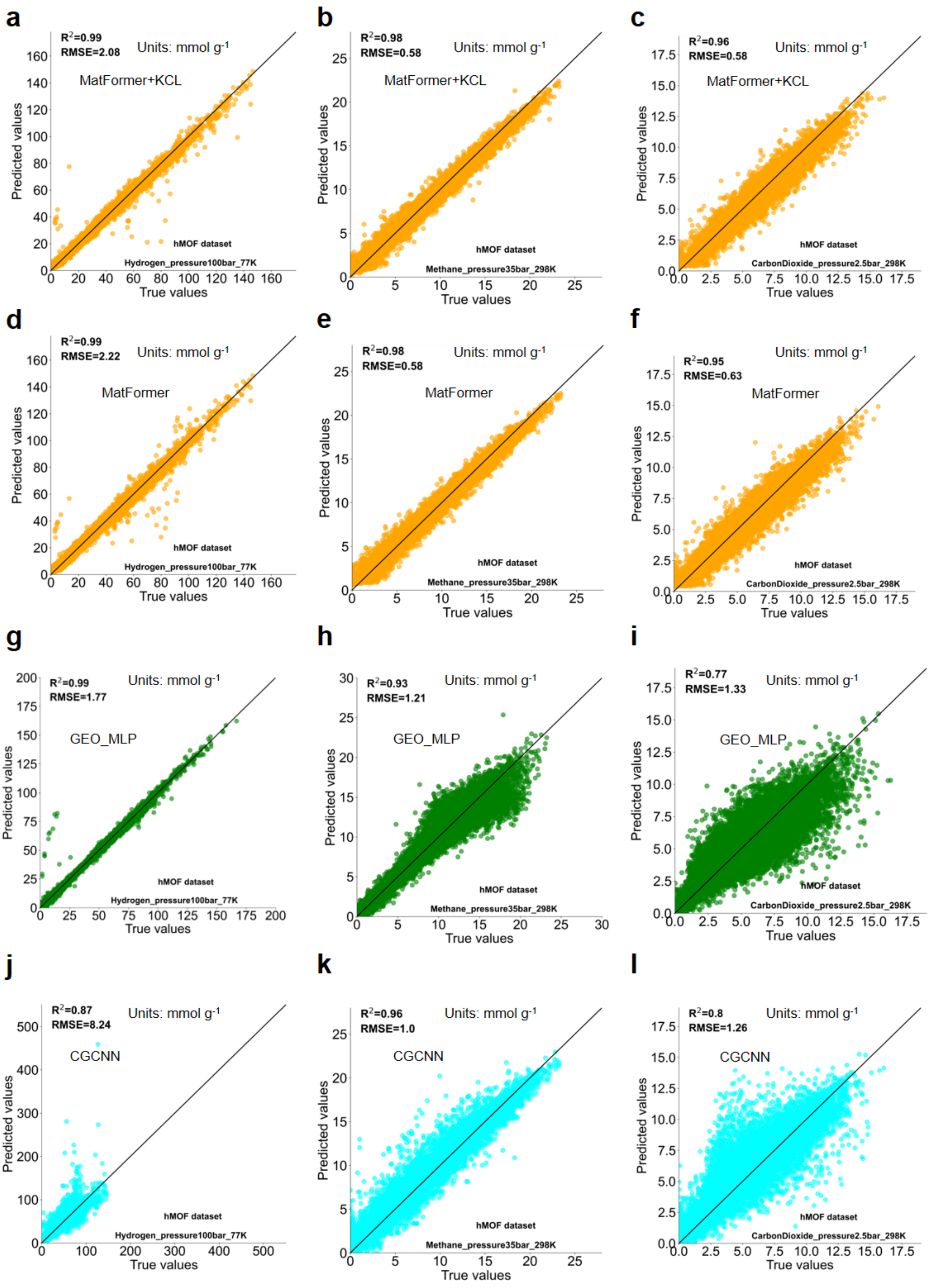

Fig. S30 | Prediction performance of adsorption capacity at high pressure in hMOF dataset. a, The correlations between true values and predicted values of H_2 adsorption capacity at 100 bar and 77 K on test set using DeepSorption (MatFormer+KCL) on hMOF dataset. **b,** The correlations

between true values and predicted values of CH_4 adsorption capacity at 35 bar and 298 K on test set using DeepSorption on hMOF dataset. **c,** The correlations between true values and predicted values of CO_2 adsorption capacity at 2.5 bar and 298 K on test set using DeepSorption on hMOF dataset. **d,** The correlations between true values and predicted values of H_2 adsorption capacity at 100 bar and 77 K on test set using MatFormer on hMOF dataset. **e,** The correlations between true values and predicted values of CH_4 adsorption capacity at 35 bar and 298 K on test set using MatFormer on hMOF dataset. **f,** The correlations between true values and predicted values of CO_2 adsorption capacity at 2.5 bar and 298 K on test set using MatFormer on hMOF dataset. **g,** The correlations between true values and predicted values of H_2 adsorption capacity at 100 bar and 77 K on test set using GEO_MLP on hMOF dataset. **h,** The correlations between true values and predicted values of CH_4 adsorption capacity at 35 bar and 298 K on test set using GEO_MLP on hMOF dataset. **i,** The correlations between true values and predicted values of CO_2 adsorption capacity at 2.5 bar and 298 K on test set using GEO_MLP on hMOF dataset. **j,** The correlations between true values and predicted values of H_2 adsorption capacity at 100 bar and 77 K on test set using CGCNN on hMOF dataset. **k,** The correlations between true values and predicted values of CH_4 adsorption capacity at 35 bar and 298 K on test set using CGCNN on hMOF dataset. **l,** The correlations between true values and predicted values of CO_2 adsorption capacity at 2.5 bar and 298 K on test set using CGCNN on hMOF dataset.

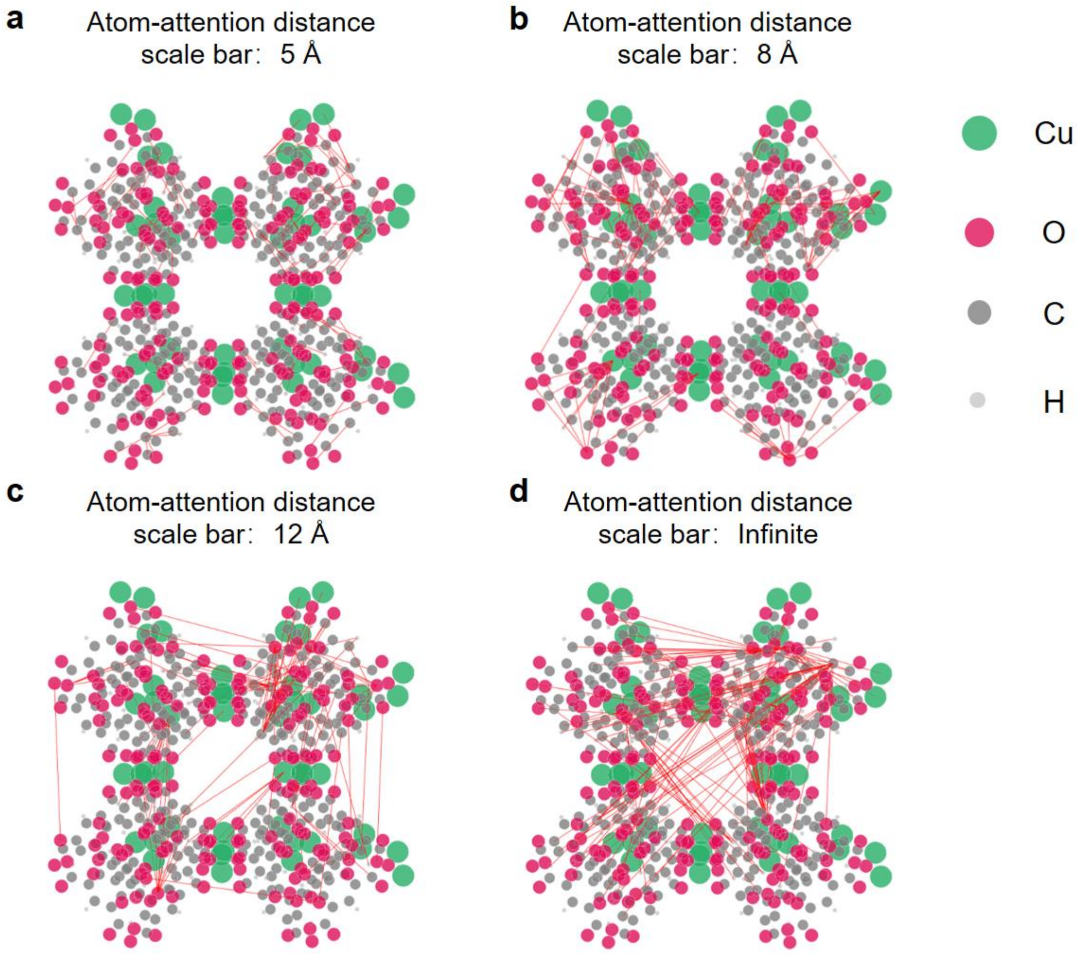

Fig. S31 | 3D attention visualization of HKUST-1. The 3D attention maps of different perspectives of HKUST-1 at different attention distance scale bars, including 5Å (**a**), 8Å (**b**), 12Å (**c**), and infinite (**d**). (Color code: C, Grey-50%; H, Grey-25%; Cu, Green; O, Pink; The attention between atom pairs, Red)

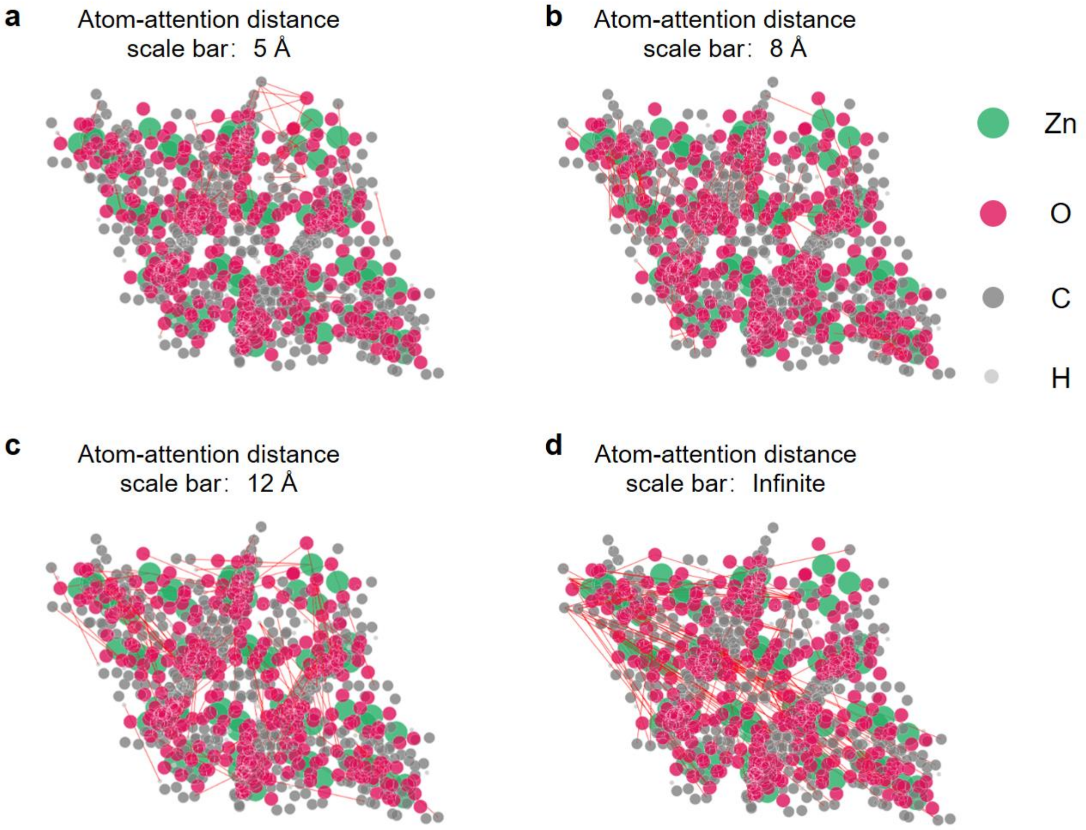

Fig. S32 | 3D attention visualization of UTSA-74. The 3D attention maps of different perspectives of UTSA-74 at different attention distance scale bars, including 5Å (**a**), 8Å (**b**), 12Å (**c**), and infinite (**d**). (Color code: C, Grey-50%; H, Grey-25%; O, Pink; Zn, Green; The attention between atom pairs, Red)

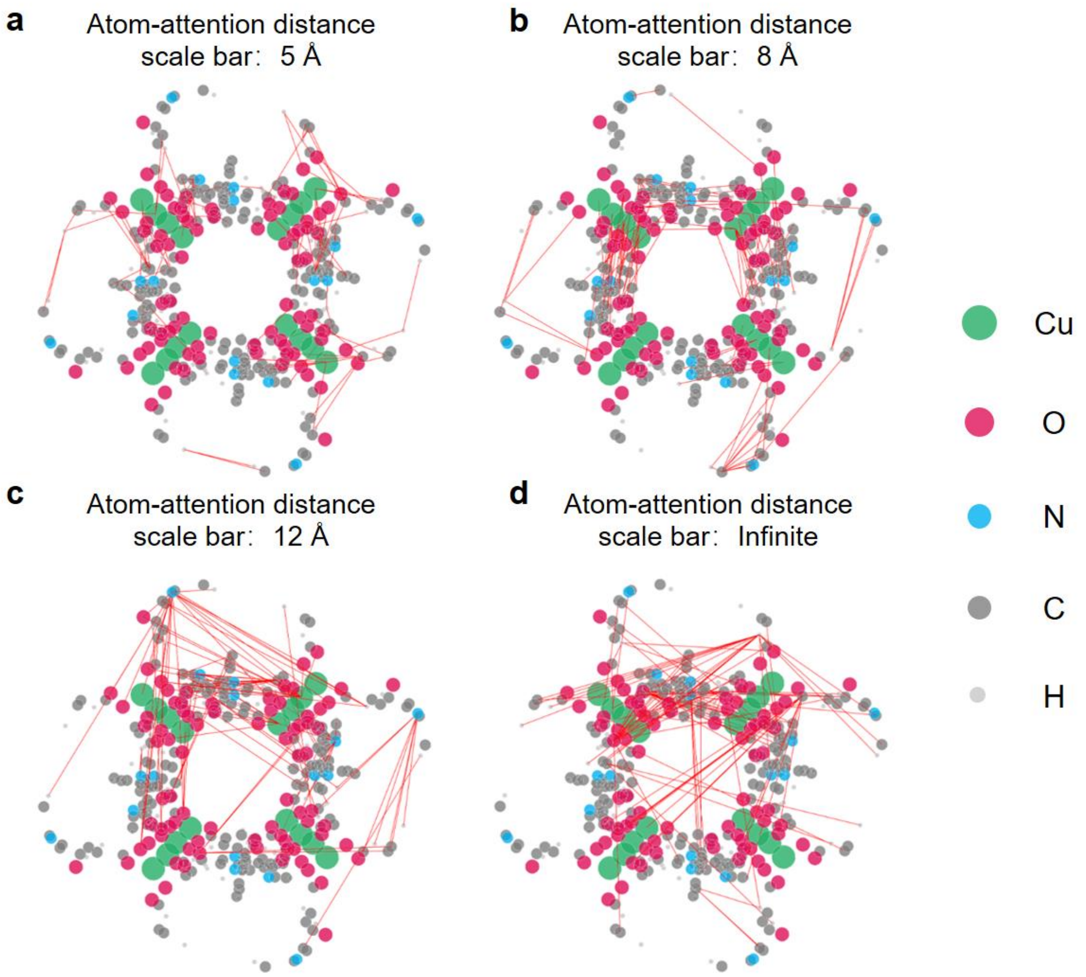

Fig. S33 | 3D attention visualization of MFM-188. The 3D attention maps of different perspectives of MFM-188 at different attention distance scale bars, including 5Å (**a**), 8Å (**b**), 12Å (**c**), and infinite (**d**). (Color code: C, Grey-50%; H, Grey-25%; N, Blue; O, Pink; Cu, Green; The attention between atom pairs, Red)

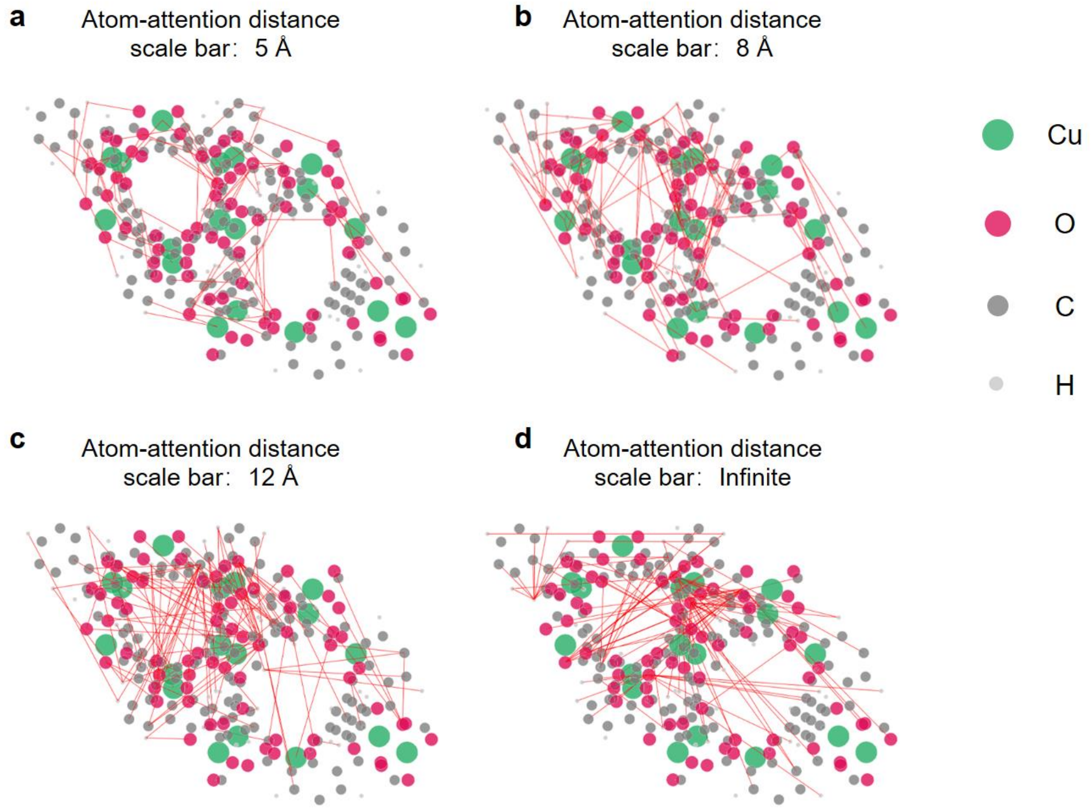

Fig. S34 | 3D attention visualization of MOF-505. The 3D attention maps of different perspectives of MOF-505 at different attention distance scale bars, including 5Å (**a**), 8Å (**b**), 12Å (**c**), and infinite (**d**). (Color code: C, Grey-50%; H, Grey-25%; Cu, Green; O, Pink; The attention between atom pairs, Red)

Supplementary Tables

	R^2	MAE	RMSE
MOFNet	0.43	20.32	28.67
SOAP_MLP	-0.08	30.05	39.45
GEO_MLP	0.33	21.78	31.03
MBTR_MLP	0.40	21.54	29.38
MBTR_MLP+KCL	0.39	21.70	29.49
CGCNN	0.48	18.94	27.28
CGCNN+KCL	0.46	19.08	27.90
RAC_MLP	0.51	18.64	26.65
RAC_MLP+KCL	0.51	19.04	26.67
LSTM	0.45	20.00	26.80
LSTM+KCL	0.55	17.70	24.30
Matformer	0.46	21.02	27.91
DeepSorption	0.70	14.39	20.73

Table S1 | Prediction performance of CO_2 adsorption capacity in CoREMOF dataset using different models. The calculated R^2 (Coefficient of determination), MAE (Mean Absolute Error), RMSE (Root Mean Square Error) of CO_2 adsorption capacity prediction on test set in CoREMOF dataset using different models to evaluate their prediction performance.

	R^2	MAE	RMSE
GEO_MLP	0.64	0.177	0.32
CGCNN	0.80	0.135	0.24
LSTM	0.79	0.141	0.25
LSTM+KCL	0.86	0.111	0.20
Matformer	0.90	0.093	0.17
DeepSorption	0.92	0.087	0.15

Table S2 | Prediction performance of CO_2 adsorption capacity in hMOF dataset using different models. The calculated R^2 (Coefficient of determination), MAE (Mean Absolute Error), RMSE (Root Mean Square Error) of CO_2 adsorption capacity prediction on test set in hMOF dataset using different models to evaluate their prediction performance.

	R^2	MAE	RMSE
GEO_MLP	0.72	0.027	0.038
CGCNN	0.86	0.019	0.027
LSTM	0.86	0.019	0.027
LSTM+KCL	0.88	0.017	0.025
Matformer	0.90	0.016	0.022
DeepSorption	0.91	0.015	0.021

Table S3 | Prediction performance of N_2 adsorption capacity in hMOF dataset using different models. The calculated R^2 (Coefficient of determination), MAE (Mean Absolute Error), RMSE (Root Mean Square Error) of N_2 adsorption capacity prediction on test set in hMOF dataset using different models to evaluate their prediction performance.

	R^2	MAE	RMSE
CoREMOF_LCD	0.90	0.673	1.11
CoREMOF_PLD	0.84	0.573	0.95
CoREMOF_D	0.97	0.067	0.09
CoREMOF_ASA	0.93	218.274	307.52
CoREMOF_VF	0.95	0.020	0.03
CoREMOF_AV	0.91	0.049	0.12
hMOF_LCD	0.98	0.444	0.58
hMOF_PLD	0.98	0.372	0.50
hMOF_ASA	0.99	145.066	195.42
hMOF_VF	0.99	0.015	0.02
hMOF_D	0.99	0.025	0.03

Table S4 | Prediction performance of expert knowledge using DeepSorption. The calculated R^2 (Coefficient of determination), MAE (Mean Absolute Error), RMSE (Root Mean Square Error) of expert knowledge on test set in CoREMOF and hMOF dataset using DeepSorption to evaluate their prediction performance.

	R^2	MAE	RMSE
CoREMOF_LCD	0.77	1.091	1.68
CoREMOF_PLD	0.67	0.927	1.37
CoREMOF_D	0.86	0.140	0.2
CoREMOF_ASA	0.78	386.169	527.94
CoREMOF_VF	0.79	0.0388	0.05
CoREMOF_AV	0.83	0.0926	0.15
hMOF_LCD	0.97	0.569	0.8
hMOF_PLD	0.94	0.557	0.75
hMOF_ASA	0.97	190.265	269.03
hMOF_VF	0.96	0.0172	0.04
hMOF_D	0.99	0.0261	0.04

Table S5 | Prediction performance of expert knowledge using LSTM+KCL. The calculated R^2 (Coefficient of determination), MAE (Mean Absolute Error), RMSE (Root Mean Square Error) of expert knowledge on test set in CoREMOF and hMOF dataset using LSTM (Long Short-Term Memory) + KCL (knowledge co-learning) to evaluate their prediction performance.

	R^2	MAE	RMSE
CoREMOF_LCD	0.36	1.520	2.80
CoREMOF_PLD	0.13	1.203	2.23
CoREMOF_D	0.82	0.158	0.21
CoREMOF_ASA	0.68	474.031	657.46
CoREMOF_VF	0.68	0.050	0.07
CoREMOF_AV	0.44	0.128	0.30

Table S6 | Prediction performance of expert knowledge using CGCNN+KCL. The calculated R^2 (Coefficient of determination), MAE (Mean Absolute Error), RMSE (Root Mean Square Error) of expert knowledge on test set in CoREMOF dataset using CGCNN+KCL to evaluate their prediction performance.

	R^2	MAE	RMSE
CoREMOF_LCD	0.41	1.655	2.68
CoREMOF_PLD	0.32	1.211	1.97
CoREMOF_D	0.75	0.177	0.25
CoREMOF_ASA	0.52	547.992	802.12
CoREMOF_VF	0.52	0.058	0.08
CoREMOF_AV	0.47	0.154	0.29

Table S7 | Prediction performance of expert knowledge using RAC_MLP+KCL. The calculated R^2 (Coefficient of determination), MAE (Mean Absolute Error), RMSE (Root Mean Square Error) of expert knowledge on test set in CoREMOF dataset using CGCNN+KCL to evaluate their prediction performance.

	R^2	MAE	RMSE
CoREMOF_LCD	0.62	1.273	2.15
CoREMOF_PLD	0.43	1.075	1.80
CoREMOF_D	0.65	0.209	0.30
CoREMOF_ASA	0.70	446.359	628.66
CoREMOF_VF	0.72	0.045	0.06
CoREMOF_AV	0.65	0.127	0.24

Table S8 | Prediction performance of expert knowledge using MBTR_MLP +KCL. The calculated R^2 (Coefficient of determination), MAE (Mean Absolute Error), RMSE (Root Mean Square Error) of expert knowledge on test set in CoREMOF dataset using CGCNN+KCL to evaluate their prediction performance.

Experiment number	Single batchsize	Periodic distance calculate	Data augmentation (extend cell)	Knowledge co-learning	MSE	MAE	R^2
1	yes	yes	yes	no	778.8	21.02	0.458
2	no	no	no	yes	494.7	15.35	0.656
3	yes	yes	no	yes	456.8	14.77	0.682
4	yes	yes	yes	yes	429.9	14.39	0.701

Table S9 | Prediction performance of CO_2 adsorption capacity in CoREMOF dataset using DeepSorption. The calculated R^2 (Coefficient of determination), MSE (Mean Square Error), MAE (Mean Absolute Error) of CO_2 adsorption capacity prediction on test set in CoREMOF dataset using DeepSorption through different training methods to evaluate their prediction performance.

Task	MAE	R^2	RMSE
AD	21.024	0.458	27.906
AD, LCD	15.386	0.673	21.682
AD, LCD, PLD	16.404	0.632	23.006
AD, LCD, PLD, AV	14.757	0.684	21.297
AD, LCD, PLD, AV, ASA	14.343	0.708	20.472
AD, LCD, PLD, AV, ASA, D	14.080	0.704	20.616
AD, LCD, PLD, AV, ASA, D, VF	14.392	0.701	20.734

Table S10 | Prediction performance of CO_2 adsorption capacity using DeepSorption. The calculated R^2 (Coefficient of determination), MAE (Mean Absolute Error), RMSE (Root Mean Square Error) of CO_2 adsorption on test set in CoREMOF dataset using DeepSorption with different subsets of descriptors.

Experiment number	Translation	Rotation Original: [X,Y,Z]	MSE	MAE	R^2
1	no	[X,Y,Z]	488.106	15.163(0.00%)	0.660
2	no	[X,Z,Y]	487.774	15.156(0.04%)	0.661
3	no	[Y,Z,X]	488.383	15.151(0.08%)	0.660
4	no	[Y,X,Z]	488.498	15.146(0.11%)	0.660
5	no	[Z,X,Y]	486.350	15.126(0.25%)	0.662
6	no	[Z,Y,X]	485.924	15.131(0.21%)	0.662
7	yes	[X,Y,Z]	513.142	15.490(2.16%)	0.643
8	yes	[X,Z,Y]	513.428	15.494(2.18%)	0.643
9	yes	[Y,Z,X]	511.687	15.469(2.02%)	0.644
10	yes	[Y,X,Z]	511.963	15.449(1.89%)	0.644
11	yes	[Z,X,Y]	514.120	15.501(2.23%)	0.642
12	yes	[Z,Y,X]	513.306	15.508(2.28%)	0.643

Table S11 | Prediction performance of CO_2 adsorption capacity in CoREMOF dataset using DeepSorption. The calculated R^2 (Coefficient of determination), MSE (Mean Square Error), MAE (Mean Absolute Error) of CO_2 adsorption capacity prediction on test set in CoREMOF dataset with translation and rotation using DeepSorption models to evaluate their prediction performance.

Experiment number	RMSE	MAE	R^2
1	20.519	14.050	0.706
2	21.614	14.192	0.693
3	20.801	14.275	0.698
4	21.189	13.730	0.698
5	21.568	14.203	0.704
6	20.720	13.598	0.712
7	22.444	15.150	0.672
8	21.461	14.708	0.691
9	21.791	15.067	0.701
10	20.206	14.057	0.705
mean	21.231	14.303	0.698
std	0.675	0.520	0.010

Table S12 | Prediction performance of CO_2 adsorption capacity in CoREMOF dataset using DeepSorption. The calculated R^2 (Coefficient of determination), RMSE (Root Mean Square Error), MAE (Mean Absolute Error) of CO_2 adsorption capacity prediction on test set in CoREMOF dataset with different dataset divisions using DeepSorption models to evaluate their prediction performance.

Task	Method	RMSE	MAE	R^2
H_2 (100 bar and 77 K)	MatFormer+KCL	2.077	1.307	0.992
H_2 (100 bar and 77 K)	MatFormer	2.220	1.408	0.991
H_2 (100 bar and 77 K)	CGCNN	8.243	4.060	0.872
H_2 (100 bar and 77 K)	GEO_MLP	1.772	0.897	0.994
CH_4 (35 bar and 298 K)	MatFormer+KCL	0.581	0.450	0.985
CH_4 (35 bar and 298 K)	MatFormer	0.584	0.443	0.985
CH_4 (35 bar and 298 K)	CGCNN	1.004	0.677	0.955
CH_4 (35 bar and 298 K)	GEO_MLP	1.209	0.803	0.934
CO_2 (2.5 bar and 298 K)	MatFormer+KCL	0.582	0.425	0.956
CO_2 (2.5 bar and 298 K)	MatFormer	0.625	0.454	0.949
CO_2 (2.5 bar and 298 K)	CGCNN	1.257	0.848	0.796
CO_2 (2.5 bar and 298 K)	GEO_MLP	1.334	0.928	0.769

Table S13 | Prediction performance of adsorption capacity at high pressure in hMOF dataset. The calculated R^2 (Coefficient of determination), RMSE (Root Mean Square Error), MAE (Mean Absolute Error) of H_2, CH_4 and CO_2 adsorption capacity prediction on test set in hMOF dataset using different models to evaluate their prediction performance.

Abbreviation	Full Name
MOF	Metal-organic framework
COF	Covalent-organic framework
PCP	Porous coordination polymer
SBU	Secondary building unit
LCD	Largest cavity diameter
PLD	Pore limiting diameter
D	Density
AV	Accessible volume
VF	Void fraction
ASA	Accessible surface area
MSA	Multi-scale Atom-attention
KCL	Knowledge co-leaning
MSE	Mean Square Error
MAE	Mean Absolute Error
RMSE	Root Mean Square Error
CGCNN	Crystal graph convolutional neural networks
EKDL	Expert-knowledge-driven learning
LSTM	Long Short-Term Memory
MLP	Multilayer perception
GPU	Graphics processing unit
FNN	Feed-forward neural network
GEO_MLP	Geometric structure multilayer perceptron
RAC	Revised autocorrelation functions
MBTR	Many-body tensor representation
SOAP	Smooth overlap of atomic positions

Table S14 | Table of abbreviations. Full names of the abbreviations that appear in the article.

Supplementary References

1 Chen, C. *et al.* A critical review of machine learning of energy materials. *Adv. Energy Mater.* **10**, 1903242 (2020).

2 Jablonka, K. M., Ongari, D., Moosavi, S. M. & Smit, B. Big-data science in porous materials: materials genomics and machine learning. *Chem. Rev.* **120**, 8066-8129 (2020).

3 Boyd, P. G. *et al.* Data-driven design of metal-organic frameworks for wet flue gas CO_2 capture. *Nature* **576**, 253-256 (2019).

4 Pollice, R. *et al.* Data-driven strategies for accelerated materials design. *Acc. Chem. Res.* **54**, 849-860 (2021).

5 Chen, L., Lan, C., Xu, B. & Bi, K. Progress on material characterization methods under big data environment. *Adv. Compos. Hybrid Mater.* **4**, 235-247 (2021).

6 Wanyonyi, F. S., Fidelis, T. T., Mutua, G. K., Orata, F. & Pembere, A. M. S. Role of pore chemistry and topology in the heavy metal sorption by zeolites: From molecular simulation to machine learning. *Comput. Mater. Sci.* **195**, 110519 (2021).

7 Altintas, C., Altundal, O. F., Keskin, S. & Yildirim, R. Machine Learning Meets with Metal Organic Frameworks for Gas Storage and Separation. *J. Chem. Inf. Model* **61**, 2131-2146 (2021).

8 Liang, H., Jiang, K., Yan, T. A. & Chen, G. H. XGBoost: an optimal machine learning model with just structural features to discover MOF adsorbents of Xe/Kr. *ACS Omega* **6**, 9066-9076 (2021).

9 He, Y., Cubuk, E. D., Allendorf, M. D. & Reed, E. J. Metallic Metal-Organic Frameworks predicted by the combination of machine learning methods and ab initio calculations. *J. Phys. Chem. Lett.* **9**, 4562-4569 (2018).

10 Fernandez, M. & Barnard, A. S. Geometrical properties can predict CO_2 and N_2 adsorption performance of Metal-Organic Frameworks (MOFs) at low pressure. *ACS Comb. Sci.* **18**, 243-252 (2016).

11 Fanourgakis, G. S., Gkagkas, K., Tylianakis, E. & Froudakis, G. E. A universal machine learning algorithm for large-scale screening of materials. *J. Am. Chem. Soc.* **142**, 3814-3822 (2020).

12 Bucior, B. J. *et al.* Energy-based descriptors to rapidly predict hydrogen storage in metal–organic frameworks. *Mol. Syst. Des. Eng.* **4**, 162-174 (2019).

13 Qiao, Z. *et al.* Molecular fingerprint and machine learning to accelerate design of high-performance homochiral metal–organic frameworks. *AIChE J.* **67,** 17352 (2021).

14 Yao, Z. *et al.* Inverse design of nanoporous crystalline reticular materials with deep generative models. *Nat. Mach. Intell.* **3**, 76-86 (2021).

15 Eckhoff, M. & Behler, J. From molecular fragments to the bulk: development of a neural network potential for MOF-5. *J. Chem. Theory. Comput.* **15**, 3793-3809 (2019).

16 Chung, Y. G. *et al.* Computation-Ready, Experimental Metal–Organic Frameworks: a tool to enable high-throughput screening of nanoporous crystals. *Chem. Mater.* **26**, 6185-6192 (2014).

17 Kim, S. *et al.* Generative Adversarial Networks for Crystal Structure Prediction. *ACS Cent. Sci.* **6**, 1412-1420 (2020).

18 Chen, C., Ye, W., Zuo, Y., Zheng, C. & Ong, S. P. Graph ntworks as a universal machine learning framework for molecules and crystals. *Chem. Mater.* **31**, 3564-3572 (2019).

19 Wang, Q. & Zhang, L. Inverse design of glass structure with deep graph neural networks. *Nat. Commun.* **12**, 5359 (2021).

20 Korolev, V., Mitrofanov, A., Korotcov, A. & Tkachenko, V. Graph convolutional neural networks as "General-Purpose" property predictors: the universality and limits of applicability. *J. Chem. Inf. Model* **60**, 22-28 (2020).

21 Xie, T. & Grossman, J. C. Crystal graph convolutional neural networks for an accurate and interpretable prediction of material properties. *Phys. Rev. Lett.* **120**, 145301 (2018).

22 Fang, Y. *et al.* Molecular contrastive learning with chemical element knowledge graph. *AAAI* (2022).

23 Sun, Z., Deng, Z. H., Nie, J. Y. & Tang, J. Rotate: Knowledge graph embedding by relational rotation in complex space. *ICLR* (2019).

24 Hochreiter, S. & Schmidhuber., J. Long short-term memory. *Neural Comput.* (1997).

25 Chen, P. *et al.* Interpretable Graph Transformer Network for Predicting Adsorption Isotherms of Metal−Organic Frameworks. *J. Chem. Inf. Model.* **62**,5446-5456 (2022).

26 Wang, S., Zhang, Z., Dai, S. & Jiang, D.-e. Insights into CO_2/N_2 selectivity in porous carbons from deep learning. *ACS Mater. Lett.* **1**, 558-563 (2019).

27 Zhang, Z. *et al.* Prediction of carbon dioxide adsorption via deep learning. *Angew. Chem. Int. Ed.* **58**, 259-263 (2019).

28 Janet, J. P., Kulik, H. J. Resolving Transition Metal Chemical Space: Feature Selection forMachine Learning and Structure–Property Relationships. *J. Phys. Chem. A.* **121**, 8939–8954 (2017).

29 Huo, H. Y. *et al.* Unified representation of molecules and crystals for machine learning. *Mach. Learn.: Sci. Technol.* **3**, 045017 (2022).

30 Bartók, A. P.; Kondor, R.; Csányi, G. On representing chemical environments. *Phys. Rev. B.* **87**, 184115 (2013)

31 Ioannidis, E. I. *et al.* Molsimplify: A toolkit for automating discovery in inorganic chemistry. *J. Comput.Chem.* **37**, 2106−2117 (2016).

32 Himanen, L. *et al.* DScribe: Library of descriptors for machine learning in materials science. *Comput. Phys. Commun.* **247**, 106949 (2020).

33 Korolev, V. V. *et al.* Transferable and extensible machine learning-derived atomic charges for modeling hybrid nanoporous materials. *Chem. Mater.* **32**, 7822-7831 (2020).
